# NASP maintains histone H3–H4 homeostasis through two distinct H3 binding modes

**DOI:** 10.1093/nar/gkac303

**Published:** 2022-04-30

**Authors:** Hongyu Bao, Massimo Carraro, Valentin Flury, Yanhong Liu, Min Luo, Liu Chen, Anja Groth, Hongda Huang

**Affiliations:** Key Laboratory of Molecular Design for Plant Cell Factory of Guangdong Higher Education Institutes, Department of Biology, School of Life Sciences, Southern University of Science and Technology, Shenzhen 518055, China; Novo Nordisk Center for Protein Research (CPR), Faculty of Health Sciences, University of Copenhagen, Copenhagen, Denmark; Biotech Research and Innovation Centre (BRIC), Faculty of Health Sciences, University of Copenhagen, Copenhagen, Denmark; Novo Nordisk Center for Protein Research (CPR), Faculty of Health Sciences, University of Copenhagen, Copenhagen, Denmark; Biotech Research and Innovation Centre (BRIC), Faculty of Health Sciences, University of Copenhagen, Copenhagen, Denmark; Key Laboratory of Molecular Design for Plant Cell Factory of Guangdong Higher Education Institutes, Department of Biology, School of Life Sciences, Southern University of Science and Technology, Shenzhen 518055, China; Key Laboratory of Molecular Design for Plant Cell Factory of Guangdong Higher Education Institutes, Department of Biology, School of Life Sciences, Southern University of Science and Technology, Shenzhen 518055, China; Key Laboratory of Molecular Design for Plant Cell Factory of Guangdong Higher Education Institutes, Department of Biology, School of Life Sciences, Southern University of Science and Technology, Shenzhen 518055, China; Novo Nordisk Center for Protein Research (CPR), Faculty of Health Sciences, University of Copenhagen, Copenhagen, Denmark; Biotech Research and Innovation Centre (BRIC), Faculty of Health Sciences, University of Copenhagen, Copenhagen, Denmark; Key Laboratory of Molecular Design for Plant Cell Factory of Guangdong Higher Education Institutes, Department of Biology, School of Life Sciences, Southern University of Science and Technology, Shenzhen 518055, China

## Abstract

Histone chaperones regulate all aspects of histone metabolism. NASP is a major histone chaperone for H3–H4 dimers critical for preventing histone degradation. Here, we identify two distinct histone binding modes of NASP and reveal how they cooperate to ensure histone H3–H4 supply. We determine the structures of a sNASP dimer, a complex of a sNASP dimer with two H3 α3 peptides, and the sNASP–H3–H4–ASF1b co-chaperone complex. This captures distinct functionalities of NASP and identifies two distinct binding modes involving the H3 α3 helix and the H3 αN region, respectively. Functional studies demonstrate the H3 αN-interaction represents the major binding mode of NASP in cells and shielding of the H3 αN region by NASP is essential in maintaining the H3–H4 histone soluble pool. In conclusion, our studies uncover the molecular basis of NASP as a major H3–H4 chaperone in guarding histone homeostasis.

## INTRODUCTION

Histone chaperones form a protein network that participates in all aspects of histone metabolism, including histone folding, posttranslational modification, transport, nucleosome assembly and disassembly, histone recycling and turnover, storage, and degradation (1–4). Thus, histone chaperones directly or indirectly influence genome stability and integrity, gene transcription, DNA replication and repair (5–7). Histone chaperones are a diverse class of proteins lacking sequence similarity that have evolved to shield histones in a myriad of ways (1). Structural studies of histones in complex with their chaperones have thus been critical to reveal the molecular basis of histone chaperone function and instrumental for functional studies. The structure of the DAXX–H3.3–H4 complex explained DAXX’s histone H3.3 variant specificity (8,9), and structure-guided functional study of TONSL revealed unique histone chaperone and reader functions in DNA replication and repair (10). More recently, structure-guided proteomics unraveled DNAJC9′s dual histone and heat-shock co-chaperone functions (11), linking ATP-resourced protein folding to nucleosome assembly pathways. Here, we focus on the structure of the nuclear autoantigenic sperm protein (NASP) and its interaction with diverse histone substrates. NASP is a conserved H3–H4 histone chaperone with central roles in histone metabolism, yet the molecular basis of sNASP escorting and safeguarding histone substrates remain elusive.

In human, NASP has two non-allelic splicing variants, the testicular NASP (tNASP) (12) and somatic NASP (sNASP) (13). tNASP is highly expressed in testes, ovarian and transformed cells, while sNASP is ubiquitously expressed. tNASP is suggested to function as a co-chaperone of HSP90 (14) and take part in the early folding of H3–H4 dimers (15), while sNASP has more broad roles in the chaperone network (15,16). sNASP is part of a multichaperone complex containing the histone chaperones ASF1 (a and b), RbAp46/48 and the histone acetyltransferase HAT1, involved in acetylation and transport of newly synthesized H3–H4 dimers (15). This multichaperone complex buffers new soluble H3–H4 dimers accumulating during replicational stress (17,18) and might also re-acetylate evicted H3–H4 dimers (16). Notably, sNASP and tNASP are uniquely required for maintaining the soluble pool of H3–H4 dimers during the cell cycle and protect them from degradation by chaperone-mediated autophagy (19). Recently, sNASP was also proposed to maintain a soluble pool of monomeric H3 in the nucleus (20). Consistent with its role as a major H3–H4 chaperone, homozygous deletion of *NASP* gene is embryonic lethal in mice (21).

NASP is broadly distributed across eukaryotes and its histone chaperone function is largely conserved (22). The fission yeast (*Schizosaccharomyces pombe*) homologue Sim3, the Arabidopsis (*Arabidopsis thaliana*) NASP and the budding yeast (*Saccharomyces cerevisiae*) homologue Hif1p were shown to bind and chaperone histones CENPA and H3 (with and without H4) (23–25). In addition, the frog (*Xenopus laevis*) homologues NASP.S (also called N1/N2) and NASP.L (also called N1/N2-like) may store maternal H3–H4 dimers in Xenopus oocytes (26,27). NASP and its homologues share an atypical tetratricopeptide repeat (TPR) domain. They constitute the SHNi-TPR (Sim3-Hif1-NASP interrupted TPR) family (23), predicted to have four TPR motifs with the TPR2 motif being interrupted by a long acidic region, a capping helix region immediately following the TPR4 motif, and a disordered C-terminal region containing a nuclear localization signal (NLS) (12,22,23,25,28,29). The recently determined structure of Hif1p mostly confirmed the structural features of the atypical SHNi-TPR domain (30). However, it has not been feasible to obtain structures of NASP family chaperones bound to histones H3 and H4 (31,32).

A variety of studies have biochemically characterized NASP and its interaction with diverse histone substrates *in vitro*. sNASP can form both a monomer and a dimer in solution (15,33), it can bind to histone H3 monomers and to H3–H4 dimers (34), and it can associate with ASF1 (a and b) to co-chaperone H3–H4 (15,35). Moreover, both sNASP and tNASP display nucleosome assembly activities toward canonical H3 and its variants (36,37). Recent work from the Ladurner lab revealed that sNASP can bind to an H3 C-terminal epitope via a central channel in its TPR domain (31), but that the chaperone uses a secondary interaction site(s) to bind H3–H4 in a co-chaperone complex with ASF1 (35). Here, we report the crystal structures of the sNASP dimer, the complex of sNASP with an H3 α3 peptide, and the sNASP–H3–H4–ASF1b co-chaperone complex. We identify two distinct H3 binding modes of NASP and our functional studies show that the H3 αN-binding mode represents the major binding mode in cells, adopted in co-chaperone complexes with ASF1 (a and b) and HAT1. Furthermore, we demonstrate that the shielding of the H3 αN region by NASP is essential in maintaining the H3–H4 histone soluble pool. In conclusion, our studies provide the molecular basis of NASP function as a major H3–H4 chaperone in maintaining histone homeostasis.

## MATERIAL AND METHODS

### Cloning and protein preparation in bacteria

The cDNA frangments of the human sNASP (amino acids, a.a. 1–340), sNASP (a.a. 30–340), sNASP (a.a. 30–340, with a deletion Δ101–159; hereafter referred to as ‘sNASP core’, sNASPc), and the budding yeast (*Saccharomyces cerevisiae*) full-length Hif1p were repectively cloned into a modified RSFDuet-1 vector (Novagen) with an N-terminal His_6_-SUMO tag. The cDNA fragments of the human full-length ASF1a, ASF1a (a.a. 1–155), ASF1b (a.a. 1–158), sNASPc, H3.3 (a.a. 1–59), and the frog (*Xenopus laevis*) NASP.S (also called N1/N2) (a.a. 23–495, with a deletion Δ97–314; hereafter referred to as ‘NASP.S core’, NASP.Sc) were repectively cloned into the pGEX-6P-1 vector (GE Healthcare) resulting an N-terminal GST-tag. We used ClonExpress II One Step Cloning Kit (Vazyme, Nanjing) to generate these clones. All mutations were introduced using a standard PCR procedure and verified by DNA sequencing. For expression of these proteins, BL21 (DE3)-RIL (Stratagene) E. coli cells were first transformed with the corresponding plasmids and then cultured using Luria-Bertani (LB) medium supplemented with proper antibiotics at 37°C to an OD_600_ of 1.0–1.2. Then, protein expression was induced with 0.5 mM isopropyl β-D-1-thiogalactopyranoside (IPTG) and cells were further incubated overnight at 20°C.

For purification of the His_6_-SUMO tagged proteins, the collected E. coli cells were suspended with buffer A (20 mM Tris pH 7.5, 1 M NaCl and 1 mM phenylmethylsulphonyl fluoride (PMSF)) and lysed by sonication. After clarification at 15,000 g for 1 hour at 4°C, the supernatants were incubated with Ni Sepharose 6 Fast Flow beads (GE Healthcare) for 1 hour at 4°C. Then the beads were washed three times with 50 mL of buffer B (20 mM Tris pH 7.5, 1 M NaCl and 40 mM imidazole). The His_6_-SUMO tagged proteins were then eluted with buffer C (20 mM Tris pH 7.5, 1 M NaCl and 500 mM imidazole). After removal of the His_6_-SUMO tag using home-made Ulp1 (SUMO protease), the proteins were further purified on a HiLoad 16/600 Superdex 200 column (GE Healthcare) with buffer D (20 mM Tris pH 7.5, 0.5 M NaCl). We noted that each of the proteins including sNASPc and its mutants, sNASP (a.a. 1–340) and sNASP (a.a. 30–340) showed two peaks corresponding to the dimer and monomer conformations on the HiLoad 16/600 Superdex 200 column, whilst the sNASPc 6E mutant only showed one peak corresponding to the monomer conformation. Moreover, the dimer:monomer ratio is not reproducible and varies a lot from one batch of purification to another.

For purification of the GST-tagged proteins, the collected E. coli cells were also suspended with buffer A (20 mM Tris pH 7.5, 1 M NaCl and 1 mM PMSF) and lysed by sonication. After clarification at 15,000 g for 1 hour at 4°C, the supernatants were incubated with Glutathione Sepharose 4B beads (GE Healthcare) for 2 hours at 4°C. Then the beads were washed three times with 50 mL of buffer A. The GST-tagged proteins were elucted with buffer E (25 mM Tris pH 7.5, 1 M NaCl and 20 mM glutathione reduced). Then the eluted proteins were further purified with a HiLoad 16/600 Superdex 200 column (GE Healthcare) in buffer D (20 mM Tris pH 7.5, 0.5 M NaCl). For some experiments, the GST-tags of GST-ASF1a (a.a. 1–155), GST-ASF1b (a.a. 1–158), GST-NASP.Sc and GST-H3.3 (a.a. 1–59) were removed with home-made 3C protease before the gel-filtration step. We noted that the GST-tagged sNASPc and its mutants and GST-tagged NASP.Sc eash showed a broad and not symmetrical peak on the HiLoad 16/600 Superdex 200 column, implicated having multiple conformations, whilst NASP.Sc with the GST-tags cut showed two peaks corresponding to the dimer and monomer conformations on the HiLoad 16/600 Superdex 200 column.

For preparation of the sNASPc-H3 α3 covalent complex, the sNASPc (a.a. 30–340, with a deletion Δ101–159) and H3.3 α3 (a.a. 116–135) fragment were covalently linked into one expression cassette, in which the H3.3 α3 fragment was fused right behind the C terminus of sNASPc without an extra linker. The resulting sNASPc-H3 α3 cassette was cloned into the modified RSFDuet-1 vector with an N-terminal His_6_-SUMO tag. The expression and purification procedures were the same as described above. We noted that the sNASPc-H3 α3 covalent complex showed two peaks corresponding to the dimer and monomer conformations (hereafter referred to as sNASPc-H3 α3 dimer and monomer, respectively) on the HiLoad 16/600 Superdex 200 column. Moreover, the dimer:monomer ratio is not reproducible and varies a lot from one batch of purification to another.

For preparation of the sNASPc-8G-ASF1b–H3–H4 heterotetramer, the sNASPc (a.a. 30–323, Δ101–159) and ASF1b histone-binding domain (HBD, a.a. 1–158) were covalently linked with a 8G-linker (GGGSGGGS) into one expression cassette, sNASPc-GGGSGGGS-ASF1b HBD (hereafter referred to as sNASPc-8G-ASF1b). The resulting sNASPc-8G-ASF1b cassette was cloned into the modified RSFDuet-1 vector with an N-terminal His_6_-SUMO tag. Then the resulting plasmid was coexpressed with a pETDuet-1 vector containing histones H3.3 and H4 genes. The resulting sNASPc-8G-ASF1b–H3–H4 heterotetramer was first purified by Ni Sepharose 6 Fast Flow beads (GE Healthcare) as above. After removal of the His_6_-SUMO tag by home-made Ulp1 (SUMO protease), the sample of protein complex was diluted with buffer F (20 mM Tris pH 7.5 only) to a final salt concerntration about 0.3 M NaCl. Then the complex was purified by a Heparin column (GE Healthcare) step, which was conducted with a salt gradient of 0.3–2.0 M NaCl. The resulting complex was further purified by a HiLoad 16/600 Superdex 200 column (GE Healthcare) with buffer D (20 mM Tris pH 7.5, 0.5 M NaCl). It should be noted that the sNASPc-8G-ASF1b–H3–H4 heterotetramer showed only one peak on the HiLoad 16/600 Superdex 200 column.

The preparation of H3.3–H4 and H3.3 (I51A R52A Y54A)–H4 tetramers followed a similar procedure in our previous study (38) with small modifications. BL21 (DE3)-RIL (Stratagene) E. coli cells were first transformed with the corresponding plasmids and then cultured using LB medium supplemented with proper antibiotics at 37°C to an OD_600_ of 1.0–1.2. Then, protein expression was induced with 1 mM IPTG and cells were further incubated for 5 hours at 37°C. The collected E. coli cells were suspended with buffer A (20 mM Tris pH 7.5, 1 M NaCl and 1 mM PMSF) and lysed by sonication. After clarification at 15,000 g for 1 hour at 4°C, the supernatants were diluted with buffer F (20 mM Tris pH 7.5 only) to a final salt concerntration about 0.5 M NaCl. The resulting supernatants were then loaded onto a heparin column followed by elution with a salt gradient of 0.5–2.0 M NaCl. The eluted fractions were analysed by SDS-PAGE. Then the pure fractions were pooled together, ajusted to a final salt concentration about 2.0 M NaCl with 5 M NaCl stock solution and concentrated. The concentrated protein samples were further purified on a HiLoad 16/600 Superdex 200 column (GE Healthcare) with a high-salt buffer G (20 mM Tris, pH 7.5, 2.0 M NaCl). The purifed histone H3–H4 complex is tetramer under this condition as reported previously (38).

### Isothermal titration calorimetry (ITC)

ITC experiments were carried out on a MicroCal PEAQ-ITC (Malvern Panalytical Ltd) at 25°C. Most of the ITC experiments had been done with the sNASPc dimer (dimer peak) and its mutants in dimer conformation (dimer peak) using buffer H (50 mM Tris pH 7.5, 200 mM NaCl), unless otherwise specified. The peptides except for the H3.3 (a.a. 1–59) peptide were all synthesized by GenScript (Nanjing). The H3.3 (a.a. 1–59) peptide was expressed in E. coli and purified as described above. The H3.3 (a.a 1–59) peptide and different sNASP proteins were buffer exchanged to buffer H (50 mM Tris pH 7.5, 200 mM NaCl). The other synthesized peptides were also dissovled in the same buffer H. For ITC assays between the H3 α3 (a.a. 116–135) peptide and different sNASP proteins, the titration protocol consisted of 19 injections of 0.4 mM H3 α3 peptide (in syringe) into 0.04 mM sNASP protein (in cell and counted as sNASP monomer). For ITC assays between the H3 N-terminal peptides, including the H3.3 (a.a. 1–59), H3.3 (a.a. 1–15), H3.3 (a.a. 16–39), H3.3 αN (a.a. 40–59), H3.3 αN R52A and H3.3 αN Y54A peptides, and different sNASP proteins, the titration protocol consisted of 19 injections of 0.8 mM peptide (in syringe) into 0.06 mM sNASP protein (in cell and counted as sNASP monomer). The concentrations of different sNASP proteins and the H3.3 (a.a. 1–59) peptide were determined by measurement of A280 using Nanodrop One (Thermo Fisher Scientific). The purities of the synthezied peptides were determined by RP-HPLC to be ≥ 95% and the weight of the synthezied peptides were determined by electronic analytical balance, of which the values were provided by GenScript (Nanjing). Then we dissolved the synthesized peptides to the working concentrations using buffer H (50 mM Tris pH 7.5, 200 mM NaCl). The data were processed with Microcal Origin software and the curves were fit to the ‘one set of sites’ model. ITC assays between the budding yeast Hifp and H3 peptides were performed in the same way.

### Crystallization

The purified sNASPc dimer at a concentration of 5 mg/ml was crystallized in 0.2 M Calcium acetate, 0.1 M HEPES, pH 7.5, 40% v/v PEG 400, with the sitting-drop vapor-diffusion method at 20°C. The concentration of PEG 400 in the mother liquor was high enough to serve as cryoprotectant, thus all crystals were directly flash frozen in liquid nitrogen. It should be noted that the sNASPc monomer could not be crystallized in our hand.

The sNASPc-H3 α3 dimer at a concentration of 3 mg/ml was crystallized in 1.4 M Na-K phosphate, pH 8.2, with the sitting-drop vapor-diffusion method at 20°C. The crystals were soaked in a cryoprotectant made form the mother liquor supplemented with 20% glycerol before being flash frozen in liquid nitrogen. It should be noted that the sNASPc-H3 α3 monomer could not be crystallized in our hand.

The sNASPc-8G-ASF1b–H3–H4 heterotetramer at a concentration of 10 mg/ml was crystallized in 8% v/v Tacsimate, pH 6.0, 20% w/v PEG 3,350, with the sitting-drop vapor-diffusion method at 20°C. The crystals were soaked in a cryoprotectant made form the mother liquor supplemented with 10% glycerol before being flash frozen in liquid nitrogen.

### X-ray Data collection and structure determination

All X-ray diffraction data were collected on beamline 19U1 of the Shanghai Synchrotron Radiation Facility (SSRF) (39). The X-ray diffraction data of the sNASPc dimer, sNASPc-H3 α3 dimer and sNASPc-8G-ASF1b–H3–H4 heterotetramer were collected at wavelengths of 0.9785 Å, 0.9766 Å and 0.9786 Å, respectively.

Data were processed and scaled using HKL3000 (40) and CCP4 program (41). The structure of the sNASPc-H3 α3 dimer was solved by molecular replacement in PHASER (42) with an inital model derived from the crystal structure of the budding yeast Hif1p (PDB 4NQ0). After molecular replacement, the strucural model was manually built using Coot (43) and refined in PHENIX (44). The structure of the sNASPc dimer was solved by molecular replacement in PHASER with the structure of the sNASPc-H3 α3 dimer as the search model, and were also manually built using Coot and refined in PHENIX. The structure of the sNASPc-8G-ASF1b–H3–H4 heterotetramer was solved by molecular replacement in PHASER with initial models derived from the structure of the sNASPc dimer and our previous structure of the MCM2–ASF1b–H3.3–H4 complex (PDB 5BNX), and were also manually built using Coot and refined in PHENIX. All the structural figures in this study were prepared with PyMOL (The PyMOL Molecular Graphics System, Schrödinger).

### GST pulldown assays

The histone H3.3–H4 tetramers were first buffer exchanged to buffer D (20 mM Tris pH 7.5, 0.5 M NaCl). For pulldowns of the GST-ASF1a–H3–H4 or GST-ASF1a–H3(I51A R52A Y54A)–H4 complexes with sNASPc and its mutants: 50 μL of Glutathione Sepharose 4B beads was suspended with 200 μL of buffer A (20 mM Tris pH 7.5, 1.0 M NaCl); and 1.5 nmol of GST-tagged full-length ASF1a (final concentration of ∼7.5 μM) and 1 nmol of H3.3–H4 tetramer or H3.3(I51A R52A Y54A)–H4 tetramer (final concentration of ∼5 μM tetramer) were added and incubated at 4°C for 1 hour; then the beads were washed quickly once with 1 mL of 1 M washing buffer (buffer A supplemented with 0.5% v/v Triton X-100), and once with 1 mL of buffer A, and twice with 1mL of buffer I (20 mM Tris pH 7.5, 0.3 M NaCl); then the beads were suspended with 200 μL of buffer I, and 1.5 nmol of purified sNASPc dimer or its mutants (dimer) (final concentration of ∼7.5 μM dimer) was added and incubated at 4°C for 1 hour; finally, the beads were washed quickly four times with 1 mL of 0.3 M washing buffer (buffer I suplemented with 0.5% v/v Triton X-100) before addition of 50 μL of SDS-PAGE sample loading buffer.

As mentioned above the GST-tagged sNASPc, frog NASP.Sc and their mutants could not be separated into dimer and monomer peaks during the purification steps, the conformations of these proteins used for pulldowns were mixtures of dimer and monomer. For pulldowns of GST-sNASPc and its mutants with the H3–H4 tetramer: 50 μL of Glutathione Sepharose 4B beads was suspended with 200 μL of buffer I (20 mM Tris pH 7.5, 0.3 M NaCl); and 1.5 nmol of GST-sNASPc or its mutants (final concentration of ∼7.5 μM) was added and incubated at 4°C for 20 min; then 1.5 nmol of purified H3.3–H4 tetramer or H3.3 (I51A R52A Y54A)–H4 tetramer (final concentration of ∼7.5 μM tetramer) was added and incubated at 4°C for another 3 hours; then the beads were washed quickly four times with 1 mL of 0.75 M washing buffer (20 mM Tris pH 7.5, 0.75 M NaCl, 0.5% v/v Triton X-100) before addition of 50 μL of SDS-PAGE sample loading buffer. Pulldowns of GST-NASP.Sc (frog) and its mutants with the H3.3–H4 tetramer, and pulldowns of GST-sNASP (30–340) and its mutants with the H3.3–H4 tetramer were performed in the same way. All the samples were analyzed with SDS-PAGE.

### Size-exclusion chromatography (SEC) assays

To check the conformational stablities of the sNASPc dimer and monomer. The purified sNASPc dimer and monomer were buffer exchanged to buffer H (50 mM Tris pH 7.5, 0.2 M NaCl). Then, 0.3 mg of the sNASPc dimer and monomer were respectively applied to a Superdex 200 Increase 10/300 GL (GE Healthcare) column pre-equilibrated with buffer H. The collected fracrions were analyzed with SDS-PAGE.

To detect the impact of the H3 αN (a.a. 40–59) and H3 α3 (a.a. 116–135) peptides on the conformations of the sNASPc dimer and monomer. First, 0.3 mg of the sNASPc dimer (final concentration 0.025 mM) or monomer (final concentration 0.05 mM) were incubated with 0.2 mM of the H3 αN or H3 α3 peptides (final concentration) in buffer H on ice for 1 hour, with a total volume of 200 μL. Then, the samples were respectively applied to a Superdex 200 Increase 10/300 GL column (GE Healthcare) pre-equilibrated with buffer H.

To reconstitute the sNASPc–H3–H4–ASF1b and sNASPc–H3–H4–ASF1a complexes *in vitro*. First, the histone H3.3–H4 tetramer was buffer exchanged to buffer D (20 mM Tris pH 7.5, 0.5 M NaCl). Then, 0.46 mg of the sNASPc dimer in buffer D was incubated with 0.44 mg of the H3.3–H4 tetramer on ice for 10 mins; later 0.40 mg of ASF1b (a.a. 1–158) or 0.40 mg of ASF1a (a.a. 1–155) was added and incubated for another 10 mins; the resulting system each with a total volume of 200 μL, with the molar ratio of sNASPc dimer: H3.3–H4 tetramer: ASF1b (or a) of 1:1:2.4. After centrifugation at 15,000 g for 10 mins, we saw a few precipitates with ASF1b but not ASF1a. Then the samples were respectively applied to a Superdex 200 Increase 10/300 GL column (GE Healthcare) pre-equilibrated with buffer D. Similar experimental settings were repeated with proteins incubated in a different order, in which ASF1b (or a) was incubated with the H3.3–H4 tetramer first, then with addition of the sNASPc dimer. The resulting samples were also analyzed by SEC assays. The collected peak fractions were analyzed with SDS-PAGE. To mesure the molar masses of the reconstituted sNASPc–H3–H4–ASF1b and sNASPc–H3–H4–ASF1a complexes, the corresponding peak fractions were collected and concentrated for the following SEC-MALS experiments.

### Size-exclusion chromatography coupled to multi-angle light scattering (SEC-MALS)

For molar mass determination, the purified proteins were analyzed using an ÄKTA-MALS system at room temperature. To determine the molar masses of the sNASPc dimer and monomer, and the 6E mutant, 100 μL solution of either proteins (concentration of 1.5 mg/mL) was injected onto a Superdex 200 Increase 10/300 GL column (GE Healthcare) equilibrated with buffer D (20 mM Tris pH 7.5, 0.5 M NaCl), at a flow rate of 0.5 mL per minute. Separation and ultraviolet detection were performed using an ÄKTA system (GE Healthcare). The ÄKTA system was coupled on-line to an 8-angle MALS detector (DAWN HELEOS II, Wyatt Technology) and a differential refractometer (Optilab T-rEX, Wyatt Technology). Data were analyzed using ASTRA 6.1.2.45. To determine the molar masses of the reconstituted sNASPc–H3–H4–ASF1b and sNASPc–H3–H4–ASF1a complexes (see above), 100 μL solution of either complexes (concentration of 1.5 mg/mL) was then applied to the SEC-MALS assay. To detect the molar mass of the sNASPc dimer–H3–H4 complex, the purified NASPc dimer was mixed with the H3–H4 tetramer at a molar ratio of 1:1, which was then applied to the SEC-MALS assay. To detect the molar mass of the sNASPc monomer–H3–H4 complex, the purified NASPc monomer was mixed with the H3–H4 tetramer at a molar ratio of 1:0.5, which was then applied to the SEC-MALS assay.

### Protein stability measurements

The stabilities of differnerent sNASPc constructs and mutants were evaluated through the inflection temperature (T_i_). The proteins were diluted into 0.1 mg/mL with 1 X PBS buffer (10 mM Na_2_HPO_4_, 1.75 mM KH_2_PO_4_, 137 mM NaCl, 2.65 mM KCl, pH 7.4) and loaded onto the standard capillaries (Cat# TY-C001; NanoTemper Technologies). The samples were measured by using Tycho NT.6 (NanoTemper Technologies). When a 30°C/min thermal ramp from 35 to 95°C was applied, the changes of the intrinsic fluorescence of protein (from residues tryptophan and tyrosine), monitored at both 350 nm and 330 nm, were recorded. The initial ratio (350 nm/330 nm at 35°C) and the change in signal over the run (Δ ratio; change between 95 and 35°C) were also recorded. The inflection temperature (T_i_) was evaluated using the Tycho NT.6 software version 1.2.2.859.

### Antibodies

See Table 1.

**Table 1.** Antibodies

Antibody	Dilution	Source	Catalog number
H3 pan	1:500	Abcam	ab10799
H4 pan	1:1000	Millipore	05–858
H4k5ac	1:1000	Abcam	ab51997
NASP	1:1000 WB, 1:250 IF	Abcam	ab181169
HAT1	1:1000	Abcam	ab194296
ASF1	1:1000	Groth *et al.*, 2007	N/A
tubulin	1:10000	Abcam	ab11316
actin	1:10000	Sigma	A5316
HA	1:1000	Cell signaling	3724
myc	1:1000	Millipore	05–724

### Recombinant DNA

See Table 2.

**Table 2.** Recombinant DNA

Plasmid	Source	Reference
pCMV6-sNASP-Myc-Flag (WT)	Origene	RC208783
pCMV6-sNASP-V265E I272E-L282E-V286E-I300E-L307E-Myc-Flag (6E)	This study	N/A
pLVX-TetOne-Puro	Clontech	631849
pLVX-TetOne-Puro-TwinStrep-HA	This study	N/A
pLVX-TetOne-puro-sNASP-TwinStrep-HA (sNASP-Strep-HA WT)	This study	N/A
pLVX-TetOne-puro-sNASP-E177A-W180A-D181A-TwinStrep-HA (sNASP-Strep-HA EWD3A)	This study	N/A
pLVX-TetOne-puro-sNASP-E246A-Y249A-L253A-TwinStrep-HA (sNASP-Strep-HA EYL3A)	This study	N/A
pLVX-TetOne-puro-sNASP-E177A-W180A-D181A-E246A-Y249A-L253A-TwinStrep-HA (sNASP-Strep-HA EWD3A + EYL3A)	This study	N/A
pUC57-NASP-FKBP12^F36V^	This study	N/A
pSpCas9(BB)-2A-Puro-V2.0	Addgene	#62988
pSpCas9(BB)-2A-Puro-NASP-sgRNA-V2.0	This study	N/A
pVSV	Addgene	#138479
psPAX2	Addgene	#12260
pGEX-6P-1-ASF1a	This study	N/A
pGEX-6P-1-ASF1a (1–155)	This study	N/A
pGEX-6P-1-ASF1b (1–158)	This study	N/A
pGEX-6P-1-sNASP (30–340)	This study	N/A
pGEX-6P-1-sNASP (30–340) E177A-W180A-D181A-E246A-Y249A-L253A	This study	N/A
pGEX-6P-1-sNASP (30–340) Δ101–159	This study	N/A
pGEX-6P-1-sNASP (30–340) Δ101–159 E177A-W180A-D181A	This study	N/A
pGEX-6P-1-sNASP (30–340) Δ101–159 E246A-Y249A-L253A	This study	N/A
pGEX-6P-1-sNASP (30–340) Δ101–159 E177A-W180A-D181A-E246A-Y249A-L253A	This study	N/A
pGEX-6P-1-NASP.S (23–495) Δ97–314	This study	N/A
pGEX-6P-1- NASP.S (23–495) Δ97–314 Q331A-W334A-D335A	This study	N/A
pGEX-6P-1- NASP.S (23–495) Δ97–314 E400A-Y403A-L407A	This study	N/A
pGEX-6P-1- NASP.S (23–495) Δ97–314 Q331A-W334A-D335A- E400A-Y403A-L407A	This study	N/A
pGEX-6P-1-H3.3 (1–59)	This study	N/A
pRSF-Duet-1-His_6_-SUMO-sNASP (1–340)	This study	N/A
pRSF-Duet-1-His_6_-SUMO-sNASP (30–340)	This study	N/A
pRSF-Duet-1-His_6_-SUMO-sNASP (30–340) Δ101–159	This study	N/A
pRSF-Duet-1- His_6_-SUMO-sNASP (30–340) Δ101–159 E91A-R100A	This study	N/A
pRSF-Duet-1- His_6_-SUMO-sNASP (30–340) Δ101–159 Q204A-L243A	This study	N/A
pRSF-Duet-1- His_6_-SUMO-sNASP (30–340) Δ101–159 R242A	This study	N/A
pRSF-Duet-1- His_6_-SUMO-sNASP (30–340) Δ101–159 E246A	This study	N/A
pRSF-Duet-1- His_6_-SUMO-sNASP (30–340) Δ101–159 Y249A	This study	N/A
pRSF-Duet-1- His_6_-SUMO-sNASP (30–340) Δ101–159 V265E-I272E-L282E-V286E-I300E-L307E	This study	N/A
pRSF-Duet-1- His_6_-SUMO-sNASP (30–340) Δ101–159 E211A-E215A-E217A	This study	N/A
pRSF-Duet-1- His_6_-SUMO-sNASP (30–340) Δ101–159 E400A-Y403A-L407A	This study	N/A
pRSF-Duet-1- His_6_-SUMO-sNASP (30–340) Δ101–159 Y257A-K313A	This study	N/A
pRSF-Duet-1- His_6_-SUMO-sNASP (30–340) Δ101–159 E305A-E309A	This study	N/A
pRSF-Duet-1- His_6_-SUMO-sNASP (30–340) Δ101–159 L306A	This study	N/A
pRSF-Duet-1- His_6_-SUMO-sNASP (30–340) Δ101–159 W180A	This study	N/A
pRSF-Duet-1- His_6_-SUMO-sNASP (30–340) Δ101–159 E177A-W180A-D181A-E246A	This study	N/A
pRSF-Duet-1- His_6_-SUMO-sNASP (30–340) Δ101–159 L185A-I188A	This study	N/A
pRSF-Duet-1- His_6_-SUMO-sNASP (30–340) Δ101–159 N218A-Q221A	This study	N/A
pRSF-Duet-1- His_6_-SUMO-sNASP (30–340) Δ101–159 E224A-E225A	This study	N/A
pRSF-Duet-1- His_6_-SUMO-sNASP (30–340) Δ101–159 – H3 (116–135)	This study	N/A
pRSF-Duet-1- His_6_-SUMO-sNASP (30–323) Δ101–159 – GGGSGGGS-ASF1b (1–158)	This study	N/A
pETDuet-H3.3-H4	This study	N/A
pETDuet-H3.3Δ55-H4	This study	N/A
pETDuet-H3.3-I51A-R52A-Y54A-H4	This study	N/A

### Primers

See Table 3.

**Table 3.** Primers

Name	Sequence
NASP_6E_forward_primer_round1	gcaGAAgcacagttcagcaaatctGAAgaagtcattgag
NASP_6E_reverse_primer_round1	cttcTTCagatttgctgaactgtgcTTCtgcctcatcatac
NASP_6E_forward_primer_round2	ctgtaGAAaacgagcagGAAaaggaggctgaagg
NASP_6E_reverse_primer_round2	ccttTTCctgctcgttTTCtacagccattctgttctc
NASP_6E_forward_primer_round3	gaaGAAgaggaactaaaggaactgGAAcccgaaattagagagaag
NASP_6E_reverse_primer_round3	cgggTTCcagttcctttagttcctcTTCttctttcttg
NASP_pLVX_subcloning_forward	Ccaactttccgtaccacttcctaccctcgtaaagaattc ATGGCCATGGAGTCCACAGCCAC
NASP_pLVX_subcloning_reverse	CCTCCGGAACCTCCACCaccggt ACATGCAGTGCTTTCAACTGTAGCTCCAGC
NASP_sgRNA	AAGCACTGCATGTTAAGAGG
NASP_clone_screening_forward	GCAAAGCAGAGTTTGGGTGACTTG
NASP_clone_screening_reverse	GACCGTTTTCCTTAGGAATCATTCCCC

### Plasmid construction for cellular experiments

The sNASP dimerization mutant 6E (V265E I272E L282E V286E I300E L307E) was generated by three successive rounds of site directed mutagenesis of the pCMV6-sNASP-Myc-Flag vector. The C-terminal TwinStrep-HA tag (sequence TGGGGSGGGASWSHPQFEKGGSGGGSWSHPQFEKGGYPYDVPDYA*) was synthetized and cloned (by Genscript) between the EcoRI and BamHI sites of the pLVX-TetOne-Puro vector (631849, Clontech). Subsequently, sNASP coding sequence were sub-cloned into pLVX-TetOne-puro-TwinStrep-HA by amplifying sNASP cDNA with primers that also create the homologous arms and using these PCR product as ‘mega-primer’ pair. NASP EWD3A(E177A-W180A-D181A), EYL3A (E246A-Y249A-L253A) and EWD3A + EYL3A (E177A-W180A-D181A-E246A-Y249A-L253A) mutants were synthetized and cloned (by Genscript) by site directed mutagenesis of pLVX-TetOne-puro-sNASP-TwinStrep-HA plasmid.

The pSpCas9(BB)-2A-Puro-NASP-sgRNA-V2.0, targeting the endogenous alleles of NASP in HCT116, was cloned between Bbsl sites of the pSpCas9(BB)-2A-Puro-V2.0 (62988, Addgene). The plasmid pUC57-NASP-FKBP12^F36V^, carrying homology arms for NASP alleles, was synthetized, and cloned by Genescript and used as a donor plasmid for genome editing.

Site directed mutagenesis was performed using established QuickChange mutagenesis protocols (Stratagene) or Infusion HD-directed mutagenesis (Takara). For Infusion HD-directed mutagenesis template plasmids were amplified with Phusion HF (F530S, Thermo Scientific) using mutagenic primers that also create homologous arms which, after PCR purification (28104, QIAgen) and Dpn1 digest (R0176L, NEB), were recombined through Infusion HD cloning (638933, Takara).

### Cell culture

Unless stated otherwise cells were grown in DMEM with Glutamax (35050061, Gibco), 10% FBS (12389802, Hyclone) and 1% penicillin/streptomycin (15140122, Gibco) at 37°C with 5% CO_2_. HeLa S3 and HCT116 pLVX-TetOne-Puro cell lines were grown under Puromycin selection (1 μg/ml, P8833, Sigma). In Hela S3 and HCT116 pLVX-TetOne-Puro, the expression of sNASP-TwinStrep-HA, referred as sNASP-Strep-HA above, wt and mutants was induced by treatment with 2 μg/ml Doxycycline (631311, Clontech) for 24–48 hours. HCT116 NASP-FKBP12^F36V^ (NASP-dTAG) were treated with 0.2% DMSO (67–68-5, Sigma) or 5 μM dTAG-13 (6605, Tocris) for the indicated times, to induce the degradation of endogenous NASP. In the complementation experiments, HCT116 cells were simultaneously treated with 2 μg/ml Doxycycline and either 0.2% DMSO or 5 μM dTAG-13 for 48 hours, to ensure a synchronized degradation of the endogenous protein and expression of sNASP-Strep-HA wt or mutants. For cell viability experiments, HCT116 cells were seeded at low confluency in a 24 well plate. 24 hours after seeding, cells were treated with either 0.2% DMSO or 5 μM dTAG-13 for 48 hours, and cell viability was quantified with CellTiter-Blue® Cell Viability Assay (G8080, Promega), according to the manufacturer's instructions.

### Genome editing

In HCT116 cells, the C-terminal of NASP was fused in frame with FKBP12^F36V^ (dTAG), immediately before the stop codon. Cells were plated in a 6 well plate and co-transfected with 1 μg of pUC57-NASP-FKBP12^F36V^ (by Genscript) and 2 μg of pSpCas9(BB)-2A-Puro-NASP-sgRNA_V2.0 (#62988, Addgene) using Lipofectamine 3000 (L3000001, Thermo Fisher Scientific), according to the manufacturer's instructions. 24 hours after transfection, cells were passaged to a 10 cm plate at the proper dilution to ensure single cell clones and selection media containing 1 μg/mL puromycin was added. 48 hours after the selection started, the selection media was removed and fresh media was added. From this point, single cell clones were isolated and moved in 96-well format. To isolate genomic DNA for genotyping, cells were washed twice with cold PBS, placed for 10 min at −80°C and incubated with squishing buffer (10 mM Tris-HCl, pH 8; 1 mM EDTA, 25 mM NaCl, 200 μg/ml Proteinase K) at 65°C for 1 hour. Proteinase K was inactivated by incubation at 95 °C for 10 minutes. 2 μl of this lysate was directly used for PCR reactions with 2 × Taq start master mix (M0496L, NEB), and the two NASP clone screening primers (see primer table). Amplified DNA was sequenced using the same two primers.

### Lentivirus generation and transfection

Cell lines expressing sNASP wt and mutants from pLVX-TetOne-Puro constructs were created via lentiviral transduction of HCT116 or HeLa S3 suspension cells and Puromycin selection (1 μg/ml) 20–24 hours post-transduction. The lentivirus containing supernatants were harvested and filtered through a 0.45 μm syringe 40–60 hours after co-transfection of 293FT cells with 10 μg pLVX-TetOne-Puro, 5 μg pVSV (#138479, Addgene), 8 μg psPAX2 (#12260, Addgene) using Lipofectamine 2000 (11668019, Thermo Fisher Scientific) according to the manufacturer's instructions. The presence of lentiviral particles was confirmed using Lenti-X GoStix Plus (631280, Clontech) according to the manufacturer's instructions.

### Cell extracts

For whole cell extracts, cells were washed twice in cold PBS and lysed with Laemmli sample buffer 1X (LSB 4X: Tris-HcL (200 mM, pH 6.8), SDS 4%, glycerol (40%), DTT (100 mM)) supplemented with Benzonase (0.015 volumes, 25 U/μl, Millipore, 70746) and MgCl2 (0.01 volume, 1 M). Extracts were left one hour at 37°C to digest DNA, boiled at 96°C for 20 minutes and cleared by centrifugation (16,000 g, 10 mins).

For soluble extracts, cells were washed twice with cold PBS and pelleted by centrifugation (300 g, 3 mins) at 4°C. The pellet was resuspended in ice cold NP40-NaCl buffer (300 mM NaCl, 0.05% Nonidet P40, 50 mM Tris-HCl pH 7.6, 0.1 mM EDTA, 5% glycerol) with freshly added inhibitors (NaF (5 mM) and β-Glycerolphosphate (10 mM), Phenylmethanesulfonyl fluoride (0.1 mM), Leupeptin (10 μg/ml), Pepstatin A (10 μg/ml), Trichostatin A (100 ng/ml), Na_3_VO_4_ (0.2 mM)) and left 15 minutes at 4°C before the lysate was cleared by centrifugation (11,000 g, 20 mins), transferred to a new tube and cleared again (11,000 g, 10 mins) and used directly for immunoprecipitation experiments or stored at -80°C.

### Immunoprecipitation and western blot analysis

Protein concentrations were measured using Pierce™ 660 nm Protein Assay Reagent (Thermo Scientific) or the Bradford protein assay (Bio-Rad). For immunoprecipitation, 2–3 mg of sNASP-FLAG-myc and sNASP-Strep-HA extracts were incubated for 3 hours at 4°C degrees with 50 μl of anti-Flag M2 (A2220, Sigma) or Anti-TwinStrep Mag-Strep beads (2–4090-002, Iba). After incubation, the beads were washed five times in ice-cold wash buffer (150 mM NaCl, 0.02% Nonidet P40, 50 mM Tris-HCl pH 7.6, 0.1 mM EDTA, 5% glycerol). Bound proteins were eluted with either Laemmli sample buffer 1X (LSB 4X: Tris-HCl (200 mM, pH 6.8), SDS 4%, glycerol (40%), DTT (100 mM)) or biotin elution buffer 1X (BXT buffer, 2–1042-025, Iba), respectively. Western blotting was performed as described previously (11). Western blots from three independent biological replicates were quantified using the software ImageJ, version 1.0.

### Immunofluorescence microscopy

HCTT116 wt and NASP-dTAG cells were seeded at a density of 3000 cells per well in 96 well plates (6055300, Cell carrier) and treated with either dTAG-13 (5 μM, 48 hours) or DMSO (0.2%, 48 hours). Before fixation, newly replicated DNA was labelled with EdU (20 mins, 10 μM) at 37°C. Cells were washed in ice-cold PBS, fixed (4% paraformaldehyde, 15 mins, 4°C) and washes twice in ice cold PBS. EdU staining followed the Click-iT plus Alexa647-picolyl azide protocol (Thermo Scientific) and DNA was stained with DAPI (4′,6-diamidino-2-phenylindole). Images were acquired on an Olympus ScanR high-content microscope and analysed with ScanR analysis software. Cell cycle gates were defined using mean EdU and total DAPI intensities.

### Data visualisation

Scatter and bar plots were visualised in GraphPad Prism (v9). All statistical analysis was performed in GraphPad Prism and statistical tests are detailed in figure legends.

## RESULTS

### Structural basis of sNASP dimerization

**Figure 1.**
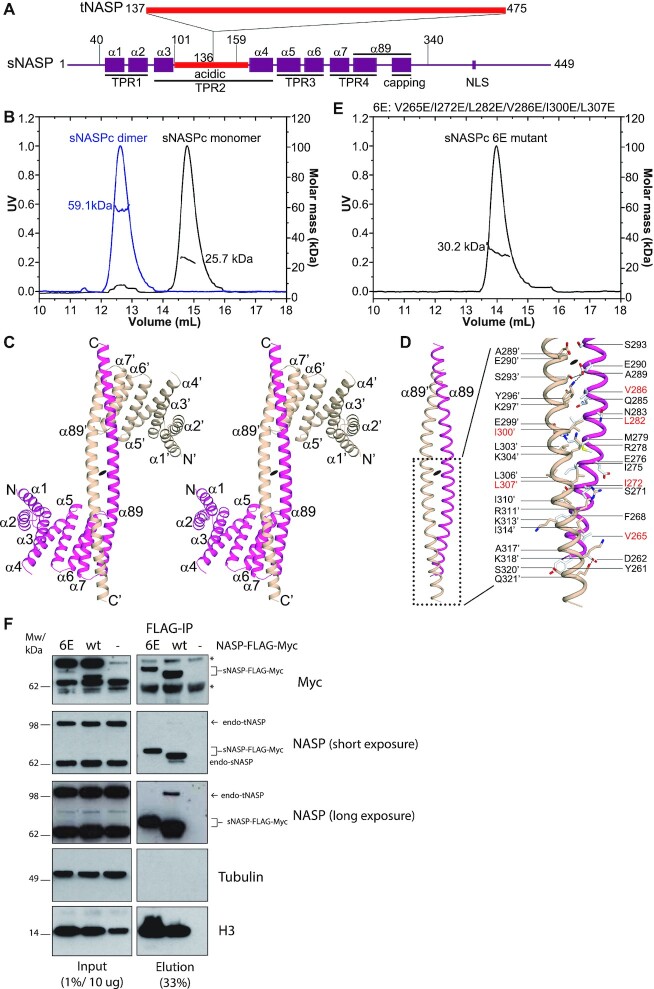
Structural and biochemical analysis of the sNASPc dimer. (**A**) Schematics of domain architectures of human sNASP and tNASP. tNASP has a longer acidic region with a 339-redidue segment inserted after position 136 of sNASP. The schematics are drawn in proportion to the number of amino acids (length). (**B**) SEC-MALS analysis of the sNASPc dimer and monomer. The purified sNASPc dimer and monomer were respectively applied to the SEC-MALS assay with a running buffer of 20 mM Tris pH 7.5, 0.5 M NaCl. The measured mass and expected mass are compared, as shown in Supplementary Table S1. (**C**) Wall-eyed stereoview of ribbon representation of the structure of the sNASPc domain-swapping dimer. The two protomers, sNASPc and sNASPc’, are colored with magenta and wheat, respectively. (**D**) Cartoon view of the antiparallel coiled-coil like structure consisting of α89 and α89′. The coiled-coil like interactions constitute of totally 66 residues (33 residues from each helix), of which the predominant interactions are hydrophobic interactions and van der Waals contacts, with a few hydrogen bonds and salt bridges further stabilizing the dimer. As the structure is related by a 2-fold symmetric axis, panel D only highlights half of the interactions between α89 and α89′. (**E**) SEC-MALS analysis of the sNASPc 6E mutant. The 6E mutant is for V265E I272E L282E V286E I300E L307E and these residues are highlighted in red in panel D. The purified sNASPc 6E mutant was applied to the SEC-MALS assay with a running buffer of 20 mM Tris pH 7.5, 0.5 M NaCl. The measured mass and expected mass are compared, as shown in Supplementary Table S1. (**F**) Immunoprecipitation of sNASP-FLAG-Myc from HeLa S3 cells transiently transfected with wt and 6E mutant sNASP constructs or untransfected control cells (−). *, unspecific band. In the second panel, all NASP proteins were detected using the anti-NASP antibody indicated in Material and Methods. The figure is a representative from two biological replicates. The second biological replicate is shown in Supplementary Figure S2E.

The human NASP has two splicing variants, sNASP and tNASP, with tNASP containing a longer acidic region (12,13). We have focused on structural studies of the TPR domain, responsible for sNASP and tNASP recognition of histones H3 and H4, and for the nucleosome assembly activity (31,36,45). To facilitate crystallization, we made a construct containing the TPR domain and capping helix region of human sNASP with the acidic region deleted (a.a 30–340 with a deletion Δ101–159; hereafter referred to as ‘sNASP core’, sNASPc) (Figure 1A and Supplementary Figure S1A). During gel-filtration purification, sNASPc was shown to have two peaks (conformations) on the chromatogram. We collected fractions from each peak and re-run the gel-filtration assays; both conformations, which were expected to be dimer and monomer, appeared to be relatively stable without obvious exchange in our experimental conditions and during the timescale of analysis (Supplementary Figure S2A,B). The dimer and monomer conformations were further corroborated by the assays of size-exclusion chromatography coupled to multi-angle light scattering (SEC-MALS) (Figure 1B and Supplementary Table S1). Interestingly, the human sNASP had been shown to homodimerize *in vitro* (15,33).

After extensive efforts, we obtained crystals from the dimeric gel-filtration fractions and solved a 3.30 Å crystal structure of sNASPc in dimer conformation (stereo view in Figure 1C and Table 4). The two protomers in the structure are related by a 2-fold symmetric axis with each protomer containing four TPR motifs. Each of the TPR1-3 motifs adopts a canonical fold, whereas the TPR4 is a variant with the predicted second helix of TPR4 and the capping region together forming a very long and extended α-helix (namely α89 here). The extended α89 is constituted of residues from Tyr261 to Gly324 (64-residues long) of sNASPc with a kink at Glu305, and the kinked α89 helix is totally about 95 Å (65 Å + 30 Å) in length (Supplementary Figure S2C). The α89 and α89′ twist one another in a left-handed manner to form an antiparallel coiled-coil like structure, with the C-terminal one third of α89′of the sNASPc’ protomer further packing against α7 and the N-terminal one third of α89 of the sNASPc protomer, and vice versa (Supplementary Figure S2D). Thus, the C-terminal one third of the α89 and α89′ helices each function like a ‘capping helix’, forming the unique architecture of a domain-swapping and dumbbell-shape dimer (Figure 1C).

**Table 4.** Data collection and refinement statistics

	sNASPc dimer (PDB 7V1K)	sNASPc-H3 α3 dimer (PDB 7V1L)	sNASPc-8G-ASF1b–H3–H4 heterotetramer (PDB 7V1M)
**Data collection**			
Space group	P6_2_22	C222_1_	P1
Cell dimensions			
*a*, *b*, *c* (Å)	95.36, 95.36, 206.23	66.90, 177.05, 65.89	71.95, 71.90, 93.43
α, β, γ (°)	90, 90, 120	90, 90, 90	70.67, 70.64, 83.61
Resolution (Å)	40.0–3.30 (3.42–3.30)^a^	40.0–2.85 (2.95–2.85)	40.0–2.85 (2.95–2.85)
*R*_pim_ (%)	3.5 (42.0)	4.5 (39.0)	6.0 (47.1)
I/*σ*(I)	17.0 (1.0)	12.7 (1.0)	10.2 (1.0)
*CC*1/2	0.518^b^	0.475	0.606
Completeness (%)	99.9 (99.8)	99.8 (98.4)	95.6 (91.3)
Redundancy	33.2 (25.9)	12.0 (10.0)	3.6 (3.3)
**Refinement**			
Resolution (Å)	39.18–3.29	36.74–2.85	35.59–2.85
No. reflections (unique)	9,000	9,478	37,598
*R*_work_ / *R*_free_ (%)	23.9/26.1	20.1/24.6	20.2/24.0^c^
No. atoms			
Protein	1,740	1,743	7,966
Peptide		109	
*B*–factors			
Protein	131.8	72.8	75.1
Peptide		73.8	
R.m.s. deviations			
Bond lengths (Å)	0.009	0.008	0.012
Bond angles (°)	0.876	0.935	1.245
Ramachandran plot			
Outliers (%)	0.0	0.0	0.0
Favored/allowed (%)	97.7/2.3	97.4/2.6	95.4/4.6

^a^Values in parentheses are for the highest-resolution shell. One crystal was used for each data set.

^b^CC1/2 of the highest-resolution shell are shown.

^c^The dataset of 7V1M was twined, and a merohedral twin law (-k, -h, -l) was applied only for the final round of refinement in PHENIX.

The coiled-coil like interactions between α89 and α89′ constitute of totally 66 residues (33 residues from each helix), of which the predominant interactions are hydrophobic interactions and van der Waals contacts, with a few hydrogen bonds and salt bridges further stabilizing the dimer (Figure 1D). Moreover, most of these interacting residues are conserved amongst different species (Supplementary Figure S1A). Consistent with the extensive interactions, many different mutants with single or even double mutations along the dimer interface failed to disrupt the dimer, and only a mutant with substitutions of six hydrophobic residues by glutamate (V265E I272E L282E V286E I300E L307E; Hereafter referred to as 6E mutant) was found to abolish the dimer formation (Figure 1E). The stability measurements revealed that the sNASPc 6E mutant had a lower inflection temperature (T_i_= 38.9°C) than the wild-type sNASPc dimer (T_i_= 58.9°C) and monomer (T_i_= 58.8°C), implicating that the sNASPc 6E mutant, though was a monomer, had a different conformation from the sNASPc monomer (Supplementary Table S2).

In accordance with our structural observations, co-immunoprecipitation experiments from HeLa S3 showed that exogenous epitope-tagged sNASP wild type (wt) pulled down both endogenous s- and t- NASP, while these interactions were impaired in the sNASP 6E mutant (Figure 1F and Supplementary Figure S2E). This supports that NASP can form both homo- and hetero- dimers *in vivo* via the α89-mediated coiled-coil like interactions. We noted that the sNASP 6E mutant was consistently expressed at a lower level than wt, suggesting that the mutations or the inability to dimerize itself affect stability. However, disrupting sNASP dimerization did not affect its histone binding *in vivo*, as both the wt and 6E mutant efficiently pulled down histone H3 (Figure 1F and Supplementary Figure S2E).

### Structural basis for sNASP recognition of H3

**Figure 2.**
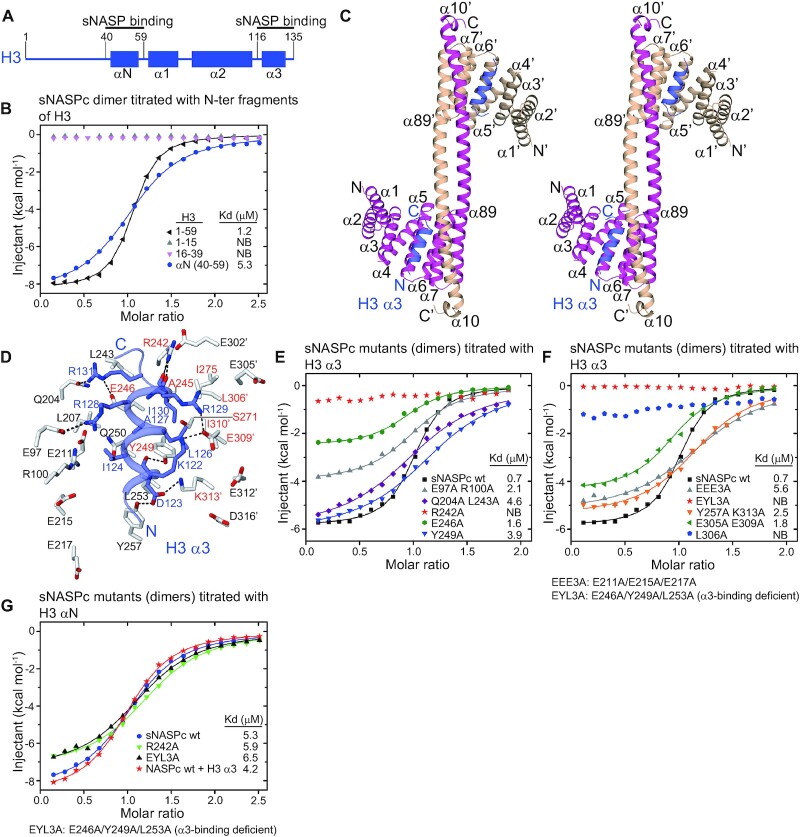
Structural and biochemical analysis of the interactions between sNASPc and H3. (**A**) Schematics of domain architecture of H3. The schematics are drawn in proportion to the number of amino acids (length). Highlighted the sNASP binding sites. (**B**) ITC analysis of the sNASPc dimer titrated with the H3 N-terminal fragments. The buffer for ITC is 50 mM Tris pH 7.5, 200 mM NaCl. The thermodynamic parameters of the ITC assays are listed in the Supplementary Table S2. All raw data from the ITC assays are shown in the Supplementary Figure S3. (**C**) Wall-eyed stereoview of ribbon representation of the structure of the sNASPc dimer in complex with two H3 α3 fragments. The two protomers of sNASPc and sNASPc’ are colored in magenta and wheat, respectively, whereas the two molecules of H3 α3 are colored in blue. (**D**) Zoom in view on the α3-binding site of the sNASPc-H3 α3 dimer structure. Interacting residues of sNASPc (white) with H3 α3 (blue) are shown in sticks representation. Hydrogen bonds are indicated by dashed blacklines. The residues consisting of the hydrophobic pocket are labeled in red. (**E-F**) ITC analysis of the sNASPc dimer and its mutants (dimers) in the α3-binding groove, titrated with the H3 α3 peptide. The buffer for ITC is 50 mM Tris pH 7.5, 200 mM NaCl. The thermodynamic parameters of the ITC assays are listed in the Supplementary Table S3. All raw data from the ITC assays are shown in the Supplementary Figure S4. (**G**) ITC analysis of the sNASPc dimer and its mutants (dimers) in the α3-binding groove and the sNASPc dimer pre-bound with H3 α3, titrated with the H3 αN peptide. The buffer for ITC is 50 mM Tris pH 7.5, 200 mM NaCl. The thermodynamic parameters of the ITC assays are listed in the Supplementary Table S2. All raw data from the ITC assays are shown in the Supplementary Figure S3.

We next undertook structural and biochemical analysis of the interactions between sNASP and H3. As a first step, our isothermal titration calorimetry (ITC) assays revealed that both the α3 region (residues 116–135) and the N-terminal region (residues 1–59) of H3 bound to the sNASPc dimer with comparable affinities at micromolar scale (*K*ds of 0.7 and 1.2 μM, respectively), and that the αN region (residues 40–59) of H3 constituted the major binding site of the N-terminal region (*K*d of 5.3 μM) (Figure 2A,B,E). As sNASPc exists as both a dimer and a monomer, we also examined the interactions of the H3 αN and α3 regions with the sNASPc monomer. Interestingly, the ITC results revealed highly similar affinities for the sNASPc dimer and monomer binding to the H3 αN peptide (*K*ds of 5.3 vs. 5.2 μM) and to the H3 α3 peptide (*K*ds of 0.7 vs. 1.0 μM) (Supplementary Figure S5A,B). Thus, the binding modes for the sNASPc dimer and monomer to interact with H3 might be similar. Our ITC results further revealed that the acidic region of sNASP was not necessary for binding to either the αN and α3 regions of H3 (Supplementary Figure S5C,D; Note: the ITC was performed with samples of a mixture of dimer and monomer). We also conducted ITC assays in different salt concentrations, 50 and 400 mM NaCl, to evaluate whether the binding affinities were modulated accordingly. The binding affinities between the sNASPc dimer and H3 αN were indeed modulated by salt concentrations, with *K*ds of 0.2, 5.3 and 14.9 μM for 50, 200 and 400 mM NaCl, respectively (Supplementary Figure S5E). A similar tendency was observed for the binding affinities between the sNASPc dimer and H3 α3, with *K*ds of 0.06, 0.7 and 1.8 μM for 50, 200 and 400 mM NaCl, respectively (Supplementary Figure S5F). This indicates that the sNASPc dimer binds to both H3 αN and α3 with sub-micromolar affinities at low salt concentrations and shows a slightly higher affinity for H3 α3 across all salt concentrations tested. It was reported that sNASP binds to a MBP-H3 (a.a. 116–135) fusion peptide with a very high affinity (reported *K*d of 0.061 μM) at 200 mM NaCl (31), while we could only observe this high affinity (*K*d of 0.06 μM) between the sNASPc dimer and H3 α3 (a.a. 116–135) peptide at 50 mM NaCl. This small inconsistency might be due to the different peptides (synthesized peptide vs. MBP fusion peptide) used for ITC assays. Interestingly, previous work showed that the N-terminal 79 residues of H3 were sufficient for binding to sNASP and tNASP in cell extracts (19), whereas another report identified an interaction between sNASP and the C-terminal α3 helix of H3 (31). Our results thus nicely corroborate and reconcile these findings. Moreover, as the sNASPc dimer and monomer appeared to be relatively stable, we used gel-filtration assays to evaluate whether they will exchange upon binding to the H3 αN and α3 peptides. The results supported that there was no obvious exchange between the sNASPc dimer and monomer even in the presence of excess H3 αN and α3 peptides (Supplementary Figure S5G,H).

To obtain further insights into the recognition of H3 by sNASP, we set out to crystallize the sNASPc dimer and monomer in the presence of the H3 αN or α3 peptides. As the initial trials were not successful, we made a covalent complex with sNASPc and the H3.3 α3 (a.a. 116–135) fragment being covalently linked into one expression cassette. The sNASPc-H3 α3 cassette was expressed and purified from E. coli. The resulting sNASPc-H3 α3 covalent complex showed two peaks corresponding to the dimer and monomer conformations (hereafter referred to as sNASPc-H3 α3 dimer and monomer, respectively) on the chromatogram during gel-filtration purification (Supplementary Figures S6A,B). After extensive crystallization trials with the sNASPc-H3 α3 dimer and monomer, we successfully solved a 2.85 Å crystal structure of the sNASPc-H3 α3 dimer (stereo view in Figure 2C). The protomers sNASPc and sNASPc’ form a dimer within the structure just as the apo sNASPc dimer structure described above, and each protomer engages an H3 α3 molecule (Figure 2C). It seems that the H3 α3 molecule bound by the protomer sNASPc is from the H3 α3 fragment fused with the other protomer sNASPc’, and vice versa (Supplementary Figure S6C). Each of the two H3 α3 molecules adopts a helical conformation highly similar to the one in the nucleosome structure and is encompassed within a groove in the concave side of each sNASPc protomer. The binding groove is formed by the conserved residues from the TPR3 and TPR4 motifs of one protomer and the ‘capping helix’ region of the other protomer, implying that dimerization is required for this binding mode. Moreover, the groove is amphipathic with a hydrophobic pocket located on one side and is surrounded by lots of conserved and negatively-charged residues (Supplementary Figure S6D,E).

The H3 α3 forms extensive interactions with the conserved residues of sNASPc that line up the groove (Figure 2D and Supplementary Figure S1A). The most prominent feature is that the main-chain carbonyl groups of H3 Arg129 and Ile130 establish three hydrogen bonds with the side chain of sNASPc Arg242, thus forming a helix-capping interactions. Another feature is that the side chains of H3 Leu126 and Ile130 are bound into the hydrophobic pocket formed by Arg242, Ala245, Glu246 and Tyr249 in NASPc α7, as well as Ser271 and Ile275 in sNASPc α8, and Leu306′, Glu309′, Ile310′ and Lys313′ from sNASPc’ α89′. It appears that the binding of H3 α3 can stabilize the sNASPc:sNASPc’ dimer. Moreover, five of the charged residues from H3 α3 contribute several salt-bridge and hydrogen-bonded interactions: both Lys122 and Arg129 of H3 establish salt bridges with Glu309′ of sNASPc’ α89′; Asp123 forms two hydrogen bonds to Tyr 249 and Tyr257 in sNASPc α7 and a salt bridge to Lys313′ in sNASPc’ α89′; Arg128 of H3 has two salt bridges with Glu97 of sNASPc α3; and Arg131 of H3 has a salt bridge and a hydrogen bond with Glu246 in sNASPc α7 and Gln204 in sNASPc α5, respectively. The van der Waals contacts among Ile124 and Ala127 of H3 with some surrounding sNASPc residues may further stabilize the interactions between H3 α3 and sNASPc. It should be noted that some of the negatively-charged residues lined up the groove, including Glu215 and Glu217 of sNASPc and Glu302′, Glu305′, Glu312′ and Asp316′ of sNASPc’, are not directly involved in binding to H3 α3 (Figure 2D).

Our ITC assays revealed that the sNASPc single mutants R242A and L306A and the triple mutant E246A Y249A L253A (EYL3A), all disrupted complex formation between the sNASPc dimer and H3 α3, whereas the other sNASPc mutants (in dimer conformation) only had intermediate effects on the binding with H3 α3 (Figure 2E,F). We focused on the mutants EYL3A, R242A and L306A that largely abolished binding. These mutants are consistent with our structural observations that Arg242 of sNASPc is involved in the prominent helix-capping interactions; Glu246, Tyr249 and Leu253 of NASPc are located at the center of the groove with Glu246 and Tyr249 further included in forming the hydrophobic pocket; Leu306′ of sNASPc’ is also involved in forming the hydrophobic pocket (Figure 2D). Interestingly, Glu246, Tyr249 and Leu253 were previously identified to be important for the interaction between sNASP and H3 α3 (31), further corroborating our structural data. As the mutants R242A, EYL3A and L306A disrupted the interactions between the sNASPc dimer and H3 α3, we went further to evaluate their folding through inflection temperature (T_i_). The sNASPc R242A dimer (T_i_= 55.4°C) and sNASPc L306A dimer (T_i_= 54.7°C) have inflection temperatures close to that of the wild-type sNASPc dimer (T_i_= 58.9°C), which implicate that they have similar structural conformations (Supplementary Table S2). The sNASPc EYL3A dimer has a lower inflection temperature (T_i_= 48.8°C) than the sNASPc dimer, suggesting that the EYL3A mutations might cause a local conformational distortion (Supplementary Table S2). Moreover, our ITC results showed that the αN region of H3 bound to the sNASPc dimer and its mutants R242A and EYL3A, as well as to the sNASPc dimer pre-bound with H3 α3, with similar affinities (Figure 2G). This revealed that the H3 α3-binding groove on the sNASPc dimer was not involved in binding to the αN region of H3 and that the sNASPc dimer can simultaneously bind to the αN and α3 regions of H3. This also indicated that the sNASPc EYL3A dimer was folded, as it bound H3 αN as efficiently as the wild-type sNASPc. Interestingly, the ITC results revealed that the sNASPc R242A monomer and sNASPc EYL3A monomer also lost interactions with H3 α3 just as their dimeric forms did (Supplementary Table S4), which further supported that the H3 α3-binding modes of the sNASPc dimer and monomer are similar.

Taken together, our structure and biochemical analysis provide the molecular basis of how sNASP engages H3.

### Structural basis for the co-chaperone relationship of sNASP and ASF1b

**Figure 3.**
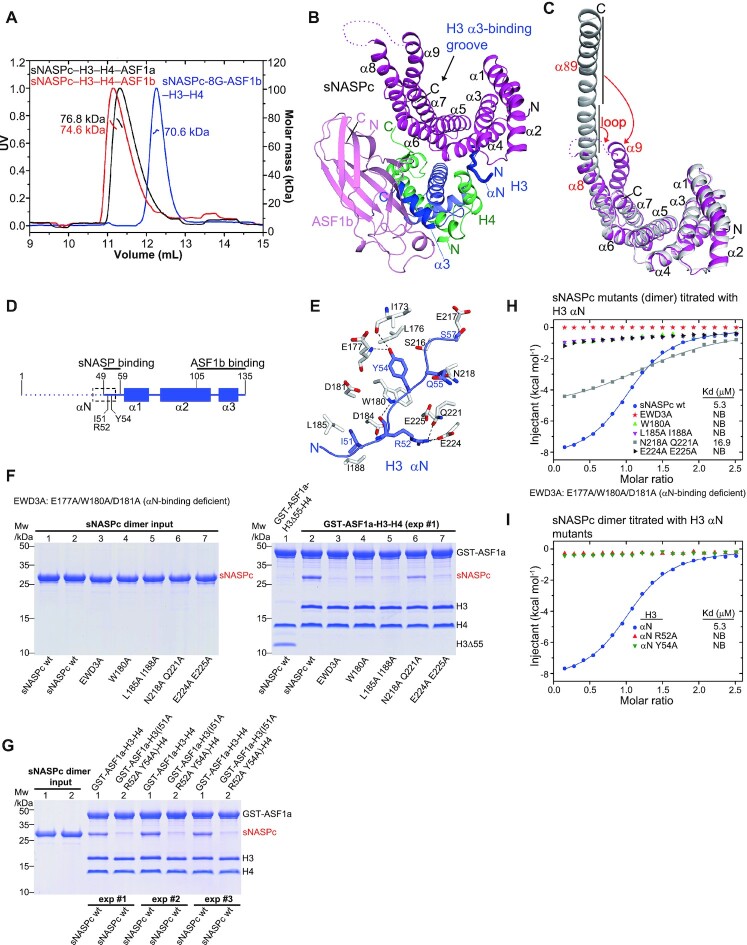
Structural and biochemical analysis of the sNASPc and ASF1b co-chaperone complex. (**A**) SEC-MALS analysis of the sNASPc–H3–H4–ASF1b and sNASPc–H3–H4–ASF1a heterotetramers, and the covalent sNASPc-8G-ASF1b–H3–H4 heterotetramer. The reconstituted sNASPc–H3–H4–ASF1b and sNASPc–H3–H4–ASF1a heterotetramers (see reconstitution in Supplementary Figure S7A,B), and the covalent sNASPc-8G-ASF1b–H3–H4 heterotetramer were respectively applied to the SEC-MALS assay with a running buffer of 20 mM Tris pH 7.5, 0.5 M NaCl. The measured mass and expected mass are compared, as shown in Supplementary Table S1. (**B**) Ribbon representation of the structure of the sNASPc-8G-ASF1b–H3–H4 heterotetramer. sNASPc, ASF1b, H3 and H4 are colored with magenta, pink, blue and green, respectively. The H3 αN region bound by sNASPc and the H3 α3 region bound by ASF1b are highlighted in dark blue. The H3 α3-binding groove observed in the sNASPc-H3 α3 dimer structure is indicated with arrow. (**C**) Structural comparisons of the sNASPc monomer of the sNASPc-8G-ASF1b–H3–H4 heterotetramer structure with one of the protomer of the sNASPc dimer structure. Highlighting the conformational changes: the central 4 turns (residues 285–298) of the long α89 helix in the dimer structure had transited into a disordered loop in the monomer structure; α89 separated into two helices, α8 and α9, in the monomer structure with α9 being folded back as a canonical capping helix. (**D**) Schematics of domain architecture of H3 in the sNASPc-8G-ASF1b–H3–H4 heterotetramer structure. Highlighted the sNASP and ASF1b binding sites. The H3 αN helix (dashed box) observed in the nucleosome structure had transited into an extended loop in the sNASPc-8G-ASF1b–H3–H4 heterotetramer structure. The residues Ile51, Arg52 and Tyr54 interacting with sNASP are indicated. The H3 N-ter not resolved in the density map of the crystal structure is indicated by dots. The schematics are drawn in proportion to the number of amino acids (length). (**E**) Zoom in view on the αN-binding site of the sNASPc-8G-ASF1b–H3–H4 heterotetramer structure. Interacting residues of sNASPc (white) with H3 αN (blue) are shown in sticks representation. Hydrogen bonds are indicated by dashed blacklines. (**F**) Pulldowns of the sNASPc dimer and its mutants (dimer) in the αN-binding site using the GST-ASF1a–H3–H4 or GST-ASF1a–H3Δ55–H4 complexes. H3Δ55 indicates H3 with a deletion of the first 55 residues, lacking the H3 αN region. For convenience, the mutants are listed in the figure. The biological replicates (exp #2 and #3) are shown in Supplementary Figure S7F. The purities of the GST-ASF1a (full-length) and H3.3–H4 tetramer used to reconstitute the GST-ASF1a–H3–H4 complex in panel F were checked by SDS-PAGE in Supplementary Figure S7G. (**G**) Pulldowns of the sNASPc dimer using the GST-ASF1a–H3–H4 or GST-ASF1a–H3 (I51A R52A Y54A)–H4 complexes. The results of exp #1 and its biological replicates (exp #2 and #3) are highly consistent. (**H**) ITC analysis of the sNASPc dimer and its mutants (dimer) in the αN-binding groove, titrated with the H3 αN peptide. The buffer for ITC is 50 mM Tris pH 7.5, 200 mM NaCl. The thermodynamic parameters of the ITC assays are listed in the Supplementary Table S2. All raw data from the ITC assays are shown in the Supplementary Figure S3. (**I**) ITC analysis of the sNASPc dimer titrated with the H3 αN peptides containing different mutation. The buffer for ITC is 50 mM Tris pH 7.5, 200 mM NaCl. The thermodynamic parameters of the ITC assays are listed in the Supplementary Table S2. All raw data from the ITC assays are shown in the Supplementary Figure S3.

sNASP is a major histone chaperone for H3–H4 dimers that cooperates with the histone chaperone ASF1 and the HAT1–RbAp46 acetyltransferase holoenzyme to maintain a soluble source of H3–H4 within the cell (15–19,46). The interactions among sNASP, H3–H4 dimers and ASF1 (a and b) had been studied through biochemical analysis (35), but the structural basis for this important and enigmatic co-chaperone complex is still elusive. To explore this, we mixed purified sNASPc dimers with H3–H4 tetramers and ASF1b (a.a. 1–158) or ASF1a (a.a. 1–155) at a molar ratio of 1:1:2.4 (sNASPc dimer: H3–H4 tetramers: ASF1) and successfully reconstituted the co-chaperone complexes, the sNASPc–H3–H4–ASF1b and sNASPc–H3–H4–ASF1a heterotetramers (Supplementary Figure S7A-C). The reconstitutions were not dependent on the mixing order of the sub-components (Supplementary Figure S7A-C). Moreover, the stoichiometry for each of the reconstituted co-chaperone complexes was 1:1:1:1 (sNASPc, H3, H4 and ASF1b or ASF1a) as confirmed by the SEC-MALS analysis (Figure 3A). This observation suggests that sNASPc adopts a monomer conformation within the sNASPc–H3–H4–ASF1b and sNASPc–H3–H4–ASF1a heterotetramers, and that sNASPc transits from dimer to monomer conformation upon binding to the H3–H4 dimer and ASF1b or ASF1a. As our attempts to crystallize the sNASPc–H3–H4–ASF1b and sNASPc–H3–H4–ASF1a heterotetramers were not successful, we constructed a covalent cassette of sNASPc-GGGSGGGS-ASF1b (a.a. 1–158) (referred to as sNASPc-8G-ASF1b) to facilitate crystallization, following similar designs used by us for structure determinations of several other co-chaperone complexes (10,38). Though with a covalent linker, the sNASPc-8G-ASF1b cassette formed a 1:1:1 complex with H3 and H4 in solution (hereafter referred to as the sNASPc-8G-ASF1b–H3–H4 heterotetramer; Figure 3A). It should point out that the covalent sNASPc-8G-ASF1b–H3–H4 heterotetramer had a different retention time on the SEC column as compared to the non-covalent sNASPc–H3–H4–ASF1b and sNASPc–H3–H4–ASF1a heterotetramers (Figure 3A). This could be due to the covalent linker within the sNASPc-8G-ASF1b cassette, which might restrain the relative motions between the sNASPc monomer and ASF1b within the co-chaperone complex.

We successfully solved a 2.85 Å crystal structure of the covalent sNASPc-8G-ASF1b–H3–H4 heterotetramer (Figure 3B), in which sNASPc indeed adopted a monomer conformation. Comparisons of the sNASPc monomer and dimer structures reveal that the central 4 turns (residues 285–298) of the long α89 helix in the dimer structure had transited into a disordered loop in the monomer structure (Figure 3C). As a result, α89 separated into two helices, α8 and α9, in the monomer structure with α9 being folded back as a canonical capping helix. The TPR1-4 motifs in the monomer structure fit well with their equivalent parts in the dimer structure, and the α9 capping helix in the monomer structure also superimposes well with the C-terminal region of the α89′ helix in the dimer structure, with an overall rmsd of 0.82 Å (Supplementary Figure S7D). Interestingly, the structure of the H3–H4–ASF1b part within the sNASPc-8G-ASF1b–H3–H4 heterotetramer is highly similar to the reported structures of the ASF1–H3–H4 heterotrimer (47,48). The α3 helix of H3 is sequestered and bound by ASF1b, thus the H3 α3-binding groove on sNASPc is unoccupied (Figure 3B), consistent with observations in a previous biochemical study (31). Moreover, sNASPc binds only to the αN region of H3 and, as expected (15,18,35), the interaction between sNASPc and ASF1b is bridged by the H3-H4 dimer (Figure 3B). Notably, the αN region of H3 adopts an extended loop conformation, also observed when H3 is bound by factors that modify or bind K56 (49,50), but distinct from the α-helix it forms within the nucleosome structure (51) (Figure 3D).

The extended αN region of H3 interacts with several conserved residues from the α4 and α6 helices and the loop connecting the α5 and α6 helices (Loop56) on the convex surface of sNASPc (Figure 3D,E and Supplementary Figure S1A). The side chain of H3 Ile51 has lots of contacts with Asp181, Asp184, Leu185 and Ile188 in sNASPc α4. The main chain carbonyl and amide of H3 Arg52 are hydrogen bonded to the side chains of Trp180 and Asp184 in sNASPc α4, respectively, while its side chain forms salt bridges and hydrogen bond with Glu224 and Gln221 in sNASPc α6, respectively, and also has contacts with Glu225 in sNASPc α6. The side chain of H3 Tyr54 penetrates into a pocket surrounded by Ile173, Leu176, Glu177 and Trp180 in NASPc α4 and Ser216 in sNASPc Loop56, with its aromatic ring establishing prominent stacking interactions with the side chain of sNASPc Trp180, and its hydroxyl group forming hydrogen bonds with the main chains of sNASPc Ile173 and Glu177. Furthermore, H3 Gln55 and Ser57 have some contacts with Asn218 and Glu217 in sNASPc Loop56, respectively.

To test the role of conserved residues for the sNASPc-H3 interactions within the sNASPc–H3–H4–ASF1b and sNASPc–H3–H4–ASF1a heterotetramers, we used the GST-ASF1a–H3–H4 complex to pull down sNASPc wt and mutants (Figure 3F and Supplementary Figure S7F). As compared to the binding of sNASPc wt, the sNASPc mutants E177A W180A D181A (EWD3A), L185A I188A, and E224A E225A disrupted interactions, while the sNASPc mutants W180A and N218A Q221A reduced binding to different extents (lanes 2–7 in Figure 3F). Concurrently, H3 with a deletion in the αN region (H3 Δ55, deletion of a.a. 1–55) disrupted interactions with sNASPc (lane 1 in Figure 3F), and the H3 mutant I51A R52A Y54A also reduced binding to sNASPc (Figure 3G). Moreover, our ITC assays with the same set of mutants showed that all of them, except the mutant sNASPc N218A Q221A, disrupted the formation of a complex between sNASPc dimer and an H3 αN peptide (Figure 3H,I). Thus, these results support our structural observations and corroborate the importance of the sNASPc-H3 αN interactions for the integrity of the sNASPc–H3–H4–ASF1b and sNASPc–H3–H4–ASF1a co-chaperone complexes. These mutants, including the sNASPc EWD3A dimer (T_i_= 61.6°C), sNASPc L185A I188A dimer (T_i_= 60.4°C), and sNASPc E224A E225A dimer (T_i_= 54.1°C), have inflection temperatures close to that of the wild-type sNASPc dimer (T_i_= 58.9°C), which implicate that they have similar structural conformations and loss of interactions are not due to protein unfolding (Supplementary Table S2). The GST pulldowns and ITC assays just mentioned had performed with the sNASPc dimer and its mutants (in dimer conformation) and identifed a key mutant, the sNASPc EWD3A dimer that lost the interaction with H3 αN. Our ITC result further revealed loss of binding of the sNASPc EWD3A monomer to H3 αN (Supplementary Table S3), suggesting that the sNASPc dimer and monomer bind to the H3 αN region in a similar manner, as shown above for the H3 α3-binding mode.

Taken together, our structure and biochemical analysis provide the molecular basis for a second histone binding mode used by NASP to co-chaperone histone H3–H4 with ASF1.

### A model for engagement of H3–H4 dimers by sNASP without ASF1

sNASP may engage H3–H4 dimers during early H3–H4 folding, and during transport, modification, and storage of histones (4,19,26). After characterizing how sNASP recognize H3 and together with ASF1b co-chaperone an H3–H4 dimer; we further explored how the dual H3 αN- and α3-binding modes of sNASP contribute to chaperoning H3–H4 dimers without ASF1.

**Figure 4.**
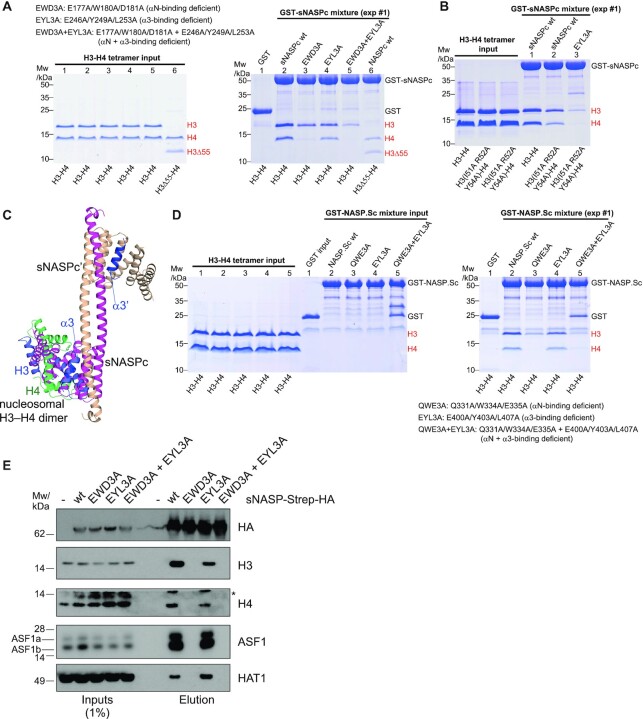
Interactions between sNASPc and H3–H4 dimers. (**A**) Pulldowns of GST-tagged sNASPc and its mutants with the H3–H4 tetramer or the H3Δ55–H4 tetramer. H3Δ55 indicates H3 with a deletion of the first 55 residues, lacking the H3 αN region. The conformations of GST-tagged sNASPc and its mutants were mixtures of dimer and monomer (referred to as GST-sNASPc mixture). The pulldowns were done by mixing the GST-sNASPc mixture and its mutants with excess H3.3–H4 tetramer or H3.3Δ55–H4 tetramer in the incubation buffer of 20 mM Tris pH 7.5, 0.3 M NaCl, and by washing with the washing buffer of 20 mM Tris pH 7.5, 0.75 M NaCl, 0.5% v/v Triton X-100. For convenience, the mutants are listed in the figure. The biological replicates (exp #2 and #3) are shown in Supplementary Figure S8A. (**B**) Pulldowns of GST-sNASPc mixture and its mutants with the H3–H4 tetramer or the H3 (I51A R52A Y54A)–H4 tetramer. H3 (I51A R52A Y54A) is a mutant with triple mutations on the H3 αN region. The pulldowns were done in the same way as panel A. The biological replicates (exp #2 and #3) are shown in Supplementary Figure S8B. (**C**) Superimposition of an H3–H4 dimer derived from the nucleosome structure (PDB 1KX5) onto the H3 α3 helix in the sNASPc-H3 α3 dimer structure. It is obvious that the nucleosomal H3–H4 dimer has lots of steric hindrances with sNASPc. The protomers NASPc and NASPc’ and the two molecules of H3 α3 are colored with magenta, wheat and dark blue respectively; the H3 and H4 from the nucleosomal H3–H4 dimer are colored with blue and green, respectively. (**D**) Pulldowns of GST-NASP.Sc mixtures and its mutants with the H3–H4 tetramer. NASP.Sc is ‘NASP.S core’, containing residues 23–495 with the acidic region (a.a. 97–314) deleted. For convenience, the mutants are listed in the figure. The pulldowns were done in the same way as panel A. The biological replicates (exp #2 and #3) are shown in Supplementary Figure S8G. (**E**) Pulldown of Strep-HA-tagged sNASP from HeLa S3 cells induced to expressed the indicated mutants or uninduced control cells (−).*, unspecific band. The figure is a representative from two biological replicates.

sNASP binds to histone H3–H4 in dimer but not tetramer conformation (15). We used GST pulldowns to study whether the H3 αN- and α3-binding sites on sNASPc were involved in its interactions with the H3–H4 dimer. As the GST-tagged sNASPc and its mutants could not be separated into dimer and monomer peaks during the purification steps, the conformations of these proteins used for GST pulldowns were mixtures of dimer and monomer. Compared to sNASPc wt, our pulldowns revealed that the sNASPc mutants EWD3A (E177A W180A D181A; H3 αN-binding deficient) and EYL3A (E246A Y249A L253A; H3 α3-binding deficient) both reduced H3–H4 binding to a similar extent, whereas the combinational mutant EWD3A + EYL3A (H3 αN- and α3-binding deficient) showed an additive effect and strongly reduced binding (Figure 4A and Supplementary Figure S8A). Concurrently, the GST-tagged sNASPc wt could pulldown the H3Δ55–H4 dimer (lacking the H3 αN region) and the H3 (I51A R52A Y54A)–H4 dimer (mutation of the H3 αN region), but much less efficiently than wild-type H3–H4 (lane 6 in Figure 4A, and Figure 4B and Supplementary Figure S8B). Moreover, the GST-tagged sNASPc EYL3A (H3 α3-binding deficient) showed strongly impaired binding to the H3 (I51A R52A Y54A)–H4 dimer (Figure 4B and Supplementary Figure S8B). These results indicate that sNASPc binds to the H3–H4 dimer via both its H3 αN- and α3-binding sites, which are respectively located on the convex and concave sides of the sNASPc TPR domain. As sNASPc did not contain the acidic region within the TPR2 motif, we made a construct sNASP (30–340) containing also the acidic region. Similarly to sNASPc, the GST-tagged sNASP (30–340) EWD3A + EYL3A (H3 αN- and α3-binding deficient) showed almost complete loss of binding to the H3–H4 dimer (Supplementary Figure S8D), further supporting that the acidic region does not influence histone binding *in vitro*. Taken together, our results support that the H3 αN- and α3-binding sites on sNASP are the major interaction sites for the H3–H4 dimer *in vitro*.

Superimposition of the canonical H3–H4 dimer structure/fold onto the H3 α3 helix bound by sNASPc shows lots of steric hindrances (Figure 4C), indicating that the H3 α3-binding mode of sNASP is not compatible with binding to an H3–H4 dimer in a canonical histone fold conformation as observed in the nucleosome structure. This argues that the H3 α3 helix would be disengaged from the histone fold to release the steric hindrances. Consistent with this idea, the GST-sNASPc EWD3A mutant, which maintains the α3-binding mode, retained more H3 than H4 in pulldowns (Figure 4A). The H3 α3-binding mode of sNASP might thus be able to destabilize the H3–H4 dimer *in vitro*, reflecting a propensity to bind H3 alone or a partially folded H3–H4 dimer. The SEC-MALS assay of the sNASPc dimer–H3–H4 complex showed a 2:2:2 complex and some higher-order complexes (Supplementary Figure S8E), whilst the SEC-MALS assay of the sNASPc monomer–H3–H4 complex only showed a 1:1:1 complex (Supplementary Figure S8F). These 2:2:2 and 1:1:1 complexes have also been observed previously (15). This suggests that the sNASP dimer and monomer can respectively engage two and one H3–H4 dimer(s) of which the H3 α3 helix might be unfolded from the histone fold. However, more structural studies will be needed to fully uncover the conformation of the H3–H4 dimer in these 2:2:2 and 1:1:1 sNASP complexes.

To address conservation of the two binding modes, we performed pulldowns with frog (*X. laevis*) NASP.S (also called N1/N2), which is a homologue of sNASP involved in storage of H3–H4 dimers in Xenopus oocytes (26). This reveals that the H3 αN- and α3-binding sites are conserved in frog NASP.S (Figure 4D and Supplementary Figure S8G). Interestingly, though the structures of sNASPc and budding yeast homologue Hif1p (30) look similar with an overall rmsd of 2.38 Å (Supplementary Figure S9A), the dual H3 αN- and α3-binding modes were not conserved in Hif1p (Supplementary Figure S9B-D), as previously suggested for the H3 α3-binding site (31). Taken together, these results thus suggest an interaction model, in which sNASP uses its H3 αN- and α3-binding sites to interact with the H3–H4 dimer *in vitro*. If the H3 α3-binding site is involved in complex formation, sNASP engages an H3–H4 dimer with the H3 α3 helix unfolded from the histone fold. This dual H3 αN- and α3-binding modes might also be implicated in H3–H4 storage by the frog NASP.S protein (also called N1/N2).

To test the importance of the dual H3 αN- and α3-binding modes for the chaperone function of sNASP *in vivo*, we performed a co-immunoprecipitation analysis of sNASP complexes from HeLa S3 cells conditionally expressing sNASP-Strep-HA wt and mutants (Figure 4E). We focused on ASF1 (a and b) and the histone acetyltransferase HAT1, both of which forms co-chaperone complexes with sNASP through mutual binding to histones (15,18). Compared to sNASP wt, the EWD3A mutant lost binding to both histones H3 and H4, ASF1 (a and b) and HAT1 (Figure 4E). In contrast, the EYL3A mutant only reduced H3 and H4 binding moderately but did not abrogate co-chaperoning with ASF1 or HAT1 binding (Figure 4E). These results validate the two independent H3 binding modes identified by our structural analysis and demonstrate that the interaction with the H3 αN region represents the major H3–H4 binding mode for sNASP in cells, and the one adopted in co-chaperone complexes with ASF1 (a and b) and HAT1. While the interaction of sNASP with the H3 α3 region is incompatible with mutual binding of ASF1 (a and b) to this site, this interaction could play a role during histone handover events where ASF1 (a and b) might transiently engage H3–H4 dimer through interaction with the H4 C-terminus.

Taken together, our results confirm the importance, both *in vitro* and *in vivo*, of the dual H3 αN- and α3-binding modes of sNASP, arguing that they both concurrently play a role in chaperoning H3 and H4 in the nucleosome assembly chain.

### Functional significance of NASP H3 αN- and α3-binding in vivo

**Figure 5.**
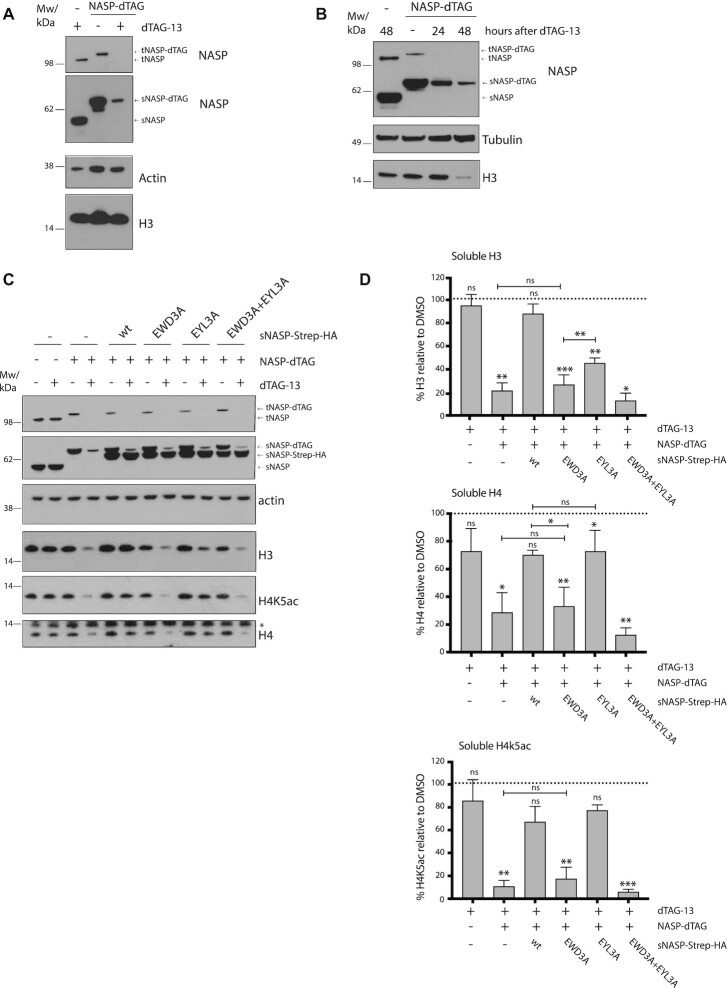
NASP maintains the soluble histone pool by chaperoning the H3 αN. (**A**) Western blot analysis of NASP in whole cell extracts of HCTT16 cells engineered to express NASP-FKBP12^F36V^ (NASP-dTAG) or wt control cells (−). Cells were treated with DMSO (−) or dTAG-13 for 48 hours as indicated. All NASP proteins were detected using the antibody indicated in Material and Methods. The figure is a representative from two biological replicates. (**B**) Western blot analysis of soluble extracts from HCT116 NASP-dTAG and control cells (−) treated as described in panel A. All NASP proteins were detected using the antibody indicated in Material and Methods. The figure is a representative from two biological replicates. (**C**) Western blot analysis of soluble extracts from HCT116 control cells (−) or NASP-dTAG (+) cells complemented as indicated with inducible expression of sNASP-Strep-HA wt or histone binding mutants. The expression of sNASP-Strep-HA was induced by DOX treatment for 48 hours and cells were co-treated with dTAG-13 for 48 hours as indicated. *, unspecific band. First and second panel, all NASP proteins were detected using the antibody indicated in Material and Methods. The figure is a representative from three biological replicates. (**D**) Quantification of H3, H4 and H4K5ac from the Western Blot analysis in panel C. The mean is shown with s.d (n = 3). Bands were quantified and normalized to actin and shown relative to DMSO treatment. P values represent unpaired two-sided t-tests (from left to right H3 quantification: P = 0.8544; 0.0095; 0.5687; 0.0010; 0.7766; 0.0019; 0.0064; 0.0138; H4 quantification: P = 0.3438; 0.0348; 0.2500; 0.0069; 0.0427; 0.6578; 0.0387; 0.1262; 0.0016; H4K5ac quantification: P = 0.8761; 0.0017; 0.0720;; 0.0023; 0.6336; 0.1359; 0.0001).

NASP is essential for maintaining a soluble pool of histones H3–H4 during the cell cycle (19). To investigate the role of the H3 αN- and α3-binding modes in this function, we generated human HCT116 cells conditional for NASP expression by integrating a degradation tag (dTAG, FKBP12^F36V^) (52) in frame with both NASP alleles (Supplementary Figure S10A). In this background, we then conditionally expressed sNASP-Strep-HA wt and histone binding mutants. Addition of the dTAG-13 molecule allowed efficient and stable depletion of endogenous s- and t-NASP isoforms, demonstrated by western blotting of whole cell extracts (Figure 5A) and immunofluorescence (Supplementary Figure S10B). In accordance with previous results (19), loss of NASP reduced the pool of soluble histone H3–H4 (Figure 5B,C) without dramatic effects on cell cycle progression and cell proliferation (Supplementary Figure S10C,D). To measure the soluble histone pool, we quantified histone H3 and H4 levels separately as well as acetylation of H4 at lysine 5 (H4K5ac), a modification introduced by HAT1 on newly synthesized histones. Conditional expression of sNASP wt rescued the soluble histone pool in NASP-dTAG cells treated with dTAG-13 molecule (Figure 5C,D), indicating that tNASP is not specifically required for maintaining the pool. The H3 αN-binding mutant (EWD3A) failed to rescue the levels of H3, H4 and H4K5ac in NASP-dTAG cells (Figure 5C,D), demonstrating that binding to the H3 αN region is essential for sNASP chaperone function to maintain the soluble histone H3–H4 pool. Intriguingly, sNASP EYL3A efficiently rescued the levels of H4 and H4K5ac, but only partially restored H3 levels. Together, and in agreeing with our structural and biochemical results, these data show that the H3 αN-binding mode is that primarily used by sNASP in chaperoning the H3–H4 histone pool, while the H3 α3-binding mode has a more specific role in regulating H3 metabolism.

## DISCUSSION

In conclusion, through structural and functional characterizations of sNASP and its interactions with histone substrates, we revealed the molecular basis of NASP as a major H3–H4 chaperone. Taken together this work supports that NASP operates through the H3 αN-binding mode to chaperone H3–H4 together with ASF1 and maintain the soluble histone H3–H4 supply. In addition, our data are consistent with the model that NASP, acting mainly upstream in the histone supply chain, chaperones monomeric H3 and partially folded H3–H4 using its H3 α3-binding mode, as biochemical data had revealed (35).

Our structures were based on the construct sNASPc, containing the core of sNASP. As tNASP is only different from sNASP with an insertion within the acidic region (Figure 1A), the structures of sNASPc are therefore likely representative of both sNASP and tNASP. We also noted there is no specific requirement for tNASP in H3–H4 chaperoning in cancer cells (our work and Cook *et al.* (19)), consistent with most somatic cells lacking this isoform. Except being rich in D/E residues, the acidic region is without any conserved motif amongst species. We found that NASP used dual binding modes for interaction with histones H3 and H4, while the acidic region of NASP was not necessary for this interaction. Interestingly, the acidic region of NASP was reported to be necessary for the interaction with linker histone H1 (45), but the structural basis of how NASP chaperones H1 are still unknown.

NASP was reported to form both a monomer and a dimer *in vitro* (33). Our sNASPc structure showed that NASP forms a domain-swapping dimer through the long α89 helix. The residues involved in dimerization are mostly conserved, at least, amongst vertebrate species. Consistently, we observed that recombinant frog NASP.S (also called N1/N2) can form both a monomer and a dimer in solution (Supplementary Figure S8H). We also found that sNASP could homodimerize, and even heterodimerize with tNASP, in human cells. This suggests sNASP and tNASP could work together in histone supply, although we note that our co-immunoprecipitations suggest that NASP homo- and hetero-dimers are not abundant in cells. The functional significance of NASP dimerization *in vivo* is still unknown. While we have shown that NASP dimerization is not required for its ability to bind histones both *in vitro* and *in vivo*, we hypothesize it plays a role in the early folding of histones by bridging together histones (H3 monomers or partially folded H3–H4 dimer) and HSP90. In this scenario, one protomer of tNASP dimer might hold histones H3 or partially folded H3–H4 and the other protomer might bind to HSP90 as a co-chaperone, which thus promotes the folding (and dimerization) of an H3–H4 dimer. We also elucidated a NASP ‘monomer’ structure, obtained in the context of the sNASPc–H3–H4–ASF1b co-chaperone complex. The NASP dimer and monomer transition involved the transformation of the long α89 helix into the α8 helix and the α9 capping helix. We envision this transition between dimer and monomer may be responsive for NASP interactions with diverse histone substrates in different cellular context. The sNASPc 6E mutant described in this study disrupts sNASP dimer formation and maintains histone binding via the H3 αN-binding mode, although having a different conformation from the wild-type sNASPc monomer (Supplementary Table S2, S3 and S4). This mutant thus provides a tool to explore the functional significance of NASP dimerization in histone supply.

Our studies provided the molecular determinants of the dual H3 α3- and αN- binding modes of NASP, of which the former was previously suggested by NMR analysis (31). The structural and biochemical results showed that both the H3 α3- and αN- binding modes of NASP were involved in binding to H3–H4 dimers and that the H3 αN-binding mode of NASP was implicated in formation of the co-chaperone complex with ASF1 (a and b) *in vitro*. In the absence of ASF1, it is unclear whether the αN- and α3-binding sites on one NASP molecule would be engaged with same H3–H4 dimer or two different H3–H4 dimers. Though we prefer the former, a crystal structure of NASP in complex with partially folded H3–H4 dimer(s) could help to resolve this in the future. Interestingly, in the absence of H3 αN-binding, sNASPc and the frog NASP.Sc retained more H3 than H4 during pulldowns. A possible explanation is that the H3 α3-binding mode of NASP might be able to destabilize the H3–H4 dimer *in vitro*, whilst the H3 αN-binding mode would stabilize the H3–H4 dimer *in vitro*. The disruption of the αN-binding mode would shift the equilibrium to destabilization of the H3–H4 dimer, explaining why this mutant retains H3 more efficiently than H4. Alternatively, it might reflect binding to the H3 monomer through the α3-helix binding mode. Our cellular data showed that the H3 αN-binding mode was the major H3–H4 binding mode for NASP, and the one adopted in co-chaperone complexes with ASF1 (a and b) and HAT1 *in vivo*. Consequently, the H3 α3-binding mode seems to have limited role in chaperoning the major H3–H4 pool *in vivo*. However, in the histone supply chain, NASP binding to the H3 α3 may be important in histone handover events between NASP and ASF1, where this H3 α3 helix could be transiently exchanged between the two chaperones. Moreover, the H3 α3-binding mode fits well with NASP function in early folding steps of the H3–H4 dimer, as NASP would hold the H3 α3 which might let the H3 α1-α2 region in a good configuration to pair with H4. Interestingly, RbAp46 binds to the α1 helix of H4 in a manner that is incompatible with histone fold formation and is thought to disengage the α1 helix of H4 from the H3–H4 core (53). The complex comprising NASP–H3–H4–RbAp46 (15,16) could thus be important early after H3–H4 synthesis, before ASF1 binding. Several proteins participate in the folding of histones H3 and H4, including the newly identified histone chaperone DNAJC9 (11) and the molecular chaperones HSC70 and HSP90 (15,46). Thus, these chaperones might compensate NASP function when it is deficient in binding to the H3 α3 region in our experiments. Interestingly, the H3 α3-binding deficient mutant, EYL3A, rescued H4/H4K5ac levels while only partially rescuing H3 levels in our complementation experiments. This represents novel evidence that a fraction of H3 in the soluble fraction exists in the form of monomer, as suggested by Apta-Smith *et al.* (20). However, we found that the H3 αN-binding mode is that primarily used by NASP in maintaining the H3–H4 dimer pool in cells, implying that shielding of the H3 N-terminus (especially the αN region) by NASP is necessary for protecting histones from degradation. ASF1 function is not required for protecting the soluble histone pool (19), underscoring that that shielding the αN region rather than co-chaperoning with ASF1 is critical in preventing degradation. This suggests that the free histone H3 tail could represent a signal for degradation or induce instability. In absence of NASP, soluble H3 and H4 are degraded by chaperone-mediated autophagy (19). The soluble histone pool is mostly composed by newly synthetized histones, with only a little contribution from evicted or old histones (54), which carry different sets of PTMs. However, following DNA damage (55,56) and transcription (57,58), the proportion of evicted histones could increase locally and it is unclear if they are also chaperoned by NASP and re-incorporated into chromatin. It would be interesting to explore whether PTMs on the H3 tail would influence its interaction with NASP and consequently the stability of evicted H3–H4 dimers. For example, the phosphorylation of H3 Tyr54 (a residue conserved in higher eukaryotes) is expected to disrupt the αN-binding mode of NASP and might thus induce H3–H4 dimer degradation. Such investigation could provide insight into whether new and evicted old histones, carrying different H3 tail PTMs, are handled by distinct histone metabolic pathways.

While this work was under evaluation, a manuscript reporting structures of NASP dimer and NASP in complex with ASF1 and histones H3–H4 was published in Genes & Development (59). The two independent studies are overall in agreement.

## DATA AVAILABILITY

Atomic coordinates and structure factors for the reported crystal structures have been deposited in the Protein Data bank under accession numbers 7V1K (the sNASPc dimer), 7V1L (the sNASPc-H3 α3 dimer), and 7V1M (the sNASPc-8G-ASF1b–H3–H4 heterotetramer). Other data are available upon reasonable request.

## Supplementary Material

gkac303_Supplemental_File
