# Supplementary material for: NASP maintains histone H3–H4 homeostasis through two distinct H3 binding modes
Source: Nucleic Acids Res. 2022 Apr 30;50(9):5349–68. doi: 10.1093/nar/gkac303 (PMC9122598; doi:10.1093/nar/gkac303)
Supplement: gkac303_Supplemental_File [file gkac303_supplemental_file.pdf]

Supplemental information for

NASP maintains histone H3–H4 homeostasis through two distinct H3 binding modes

Hongyu Bao[1], Massimo Carraro[2,3], Valentin Flury[2,3], Yanhong Liu[1], Min Luo[1], Liu Chen[1], Anja Groth[2,3,]*, Hongda Huang[1,]*

[1]Key Laboratory of Molecular Design for Plant Cell Factory of Guangdong Higher Education Institutes, Department of Biology, School of Life Sciences, Southern University of Science and Technology, Shenzhen 518055, China.

[2]Novo Nordisk Center for Protein Research (CPR), Faculty of Health Sciences, University of Copenhagen, Copenhagen, Denmark.

[3]Biotech Research and Innovation Centre (BRIC), Faculty of Health Sciences, University of Copenhagen, Copenhagen, Denmark.

*To whom correspondence should be addressed. Email: huanghd@sustech.edu.cn

Correspondence may also be addressed to Anja Groth, Email: anja.groth@cpr.ku.dk

The authors wish it to be known that, in their opinion, the first two authors should be regarded as joint First Authors

This PDF file includes:

Supplementary Figures S1 to S10
Supplementary Tables S1 to S4

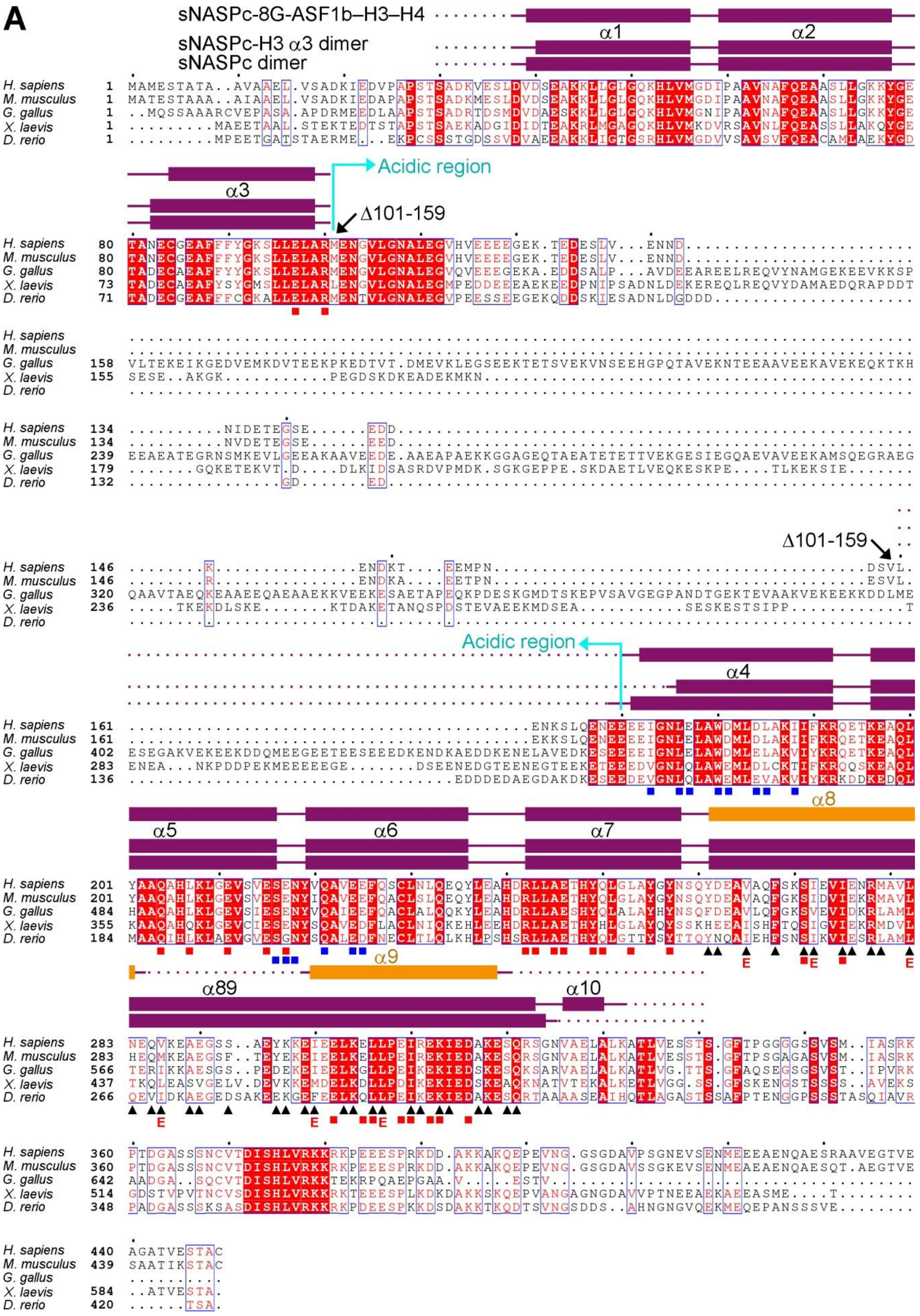

Supplementary Figure S1. Sequence alignment of NASP. (**A**) Sequence alignment of NASP from different species: *H. sapiens* sNASP (NP_689511); *M.*

musculus sNASP (NP_001074944); *G. gallus* NASP (XP_015146756); *X. laevis* NASP.S (also called N1/N2) (NP_001081537); *D. rerio* sNASP (XP_021332627). The conserved and identical residues across species are boxed and highlighted in red. Secondary structure elements derived from the structures of the sNASPc dimer, sNASPc-H3 α3 dimer and sNASPc-8G-ASF1b–H3–H4 heterotetramer are shown on top of the alignments. The disordered regions not resolved in the density maps of the crystal structures are indicated by magenta dots. Under the alignments, '▲' highlights the 33 residues in the α89 helix consisting of the dimerization interface of the sNASPc dimer; '■' highlights the 25 residues forming the H3 α3-binding groove of sNASPc; '■' highlights the 14 residues forming the H3 αN-binding site of sNASPc; 'E' highlights the 6E mutant that disrupts the sNASPc dimer.

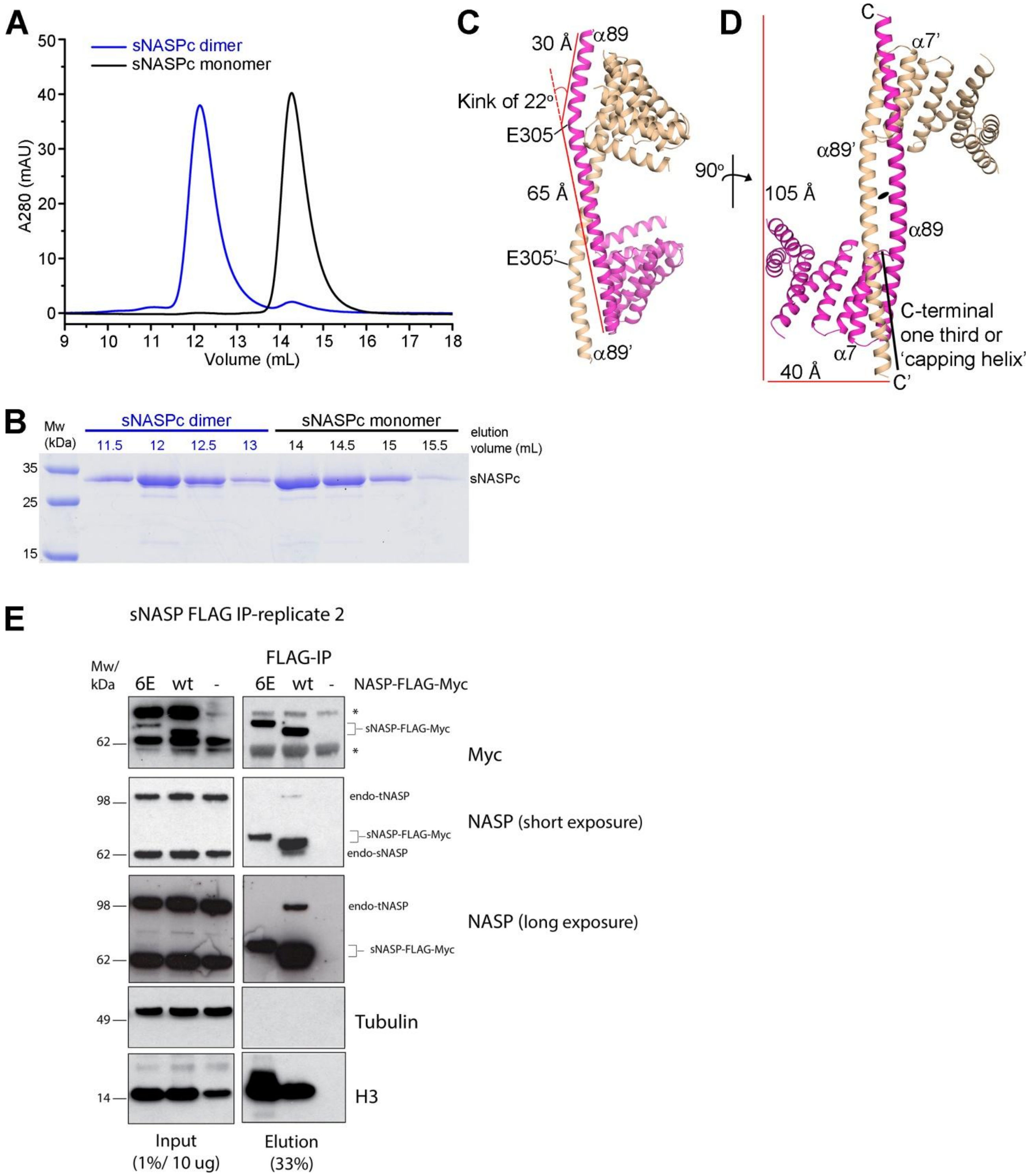

Supplementary Figure S2. Gel-filtration analysis and structure of the sNASPc dimer. (**A**) Two peaks or conformations of sNASPc were found in the gel-filtration chromatogram during purification. The fractions from each peak were collected separately and re-loaded onto the Superdex 200 Increase 10/300 GL column (GE Healthcare) for analysis. The SEC experiments were

run with the buffer of 50 mM Tris pH 7.5, 0.2 M NaCl. The two conformations are relatively stable in our experimental conditions and expected to be dimer and monomer. (**B**) The peak fractions from panel A, experiment #1 with the sNASPc dimer and experiment #2 with the sNASPc monomer, were analyzed with SDS-PAGE. The fractions were collected 0.5 mL per tube, while the numbers on top of the SDS-PAGE gel indicated the elution volume in which the fractions started. (**C-D**) Structures of the sNASPc dimer highlighting the Kink structures in the long helices α89 and α89' (**C**) and how the C-terminal one third of α89' of the sNASPc' protomer packs against α7 and the N-terminal one third of α89 of the sNASPc protomer, and vice versa (**D**). The two protomers sNASPc and sNASPc' are colored with magenta and wheat, respectively. (**E**) The second biological replicate of Figure 1F. Immunoprecipitation of sNASP-FLAG-Myc from HeLa S3 cells transiently transfected with wt and 6E mutant sNASP constructs or untransfected control cells (-). *, unspecific band. In the second panel, all NASP proteins were detected using the anti-NASP antibody indicated in Material and Methods.

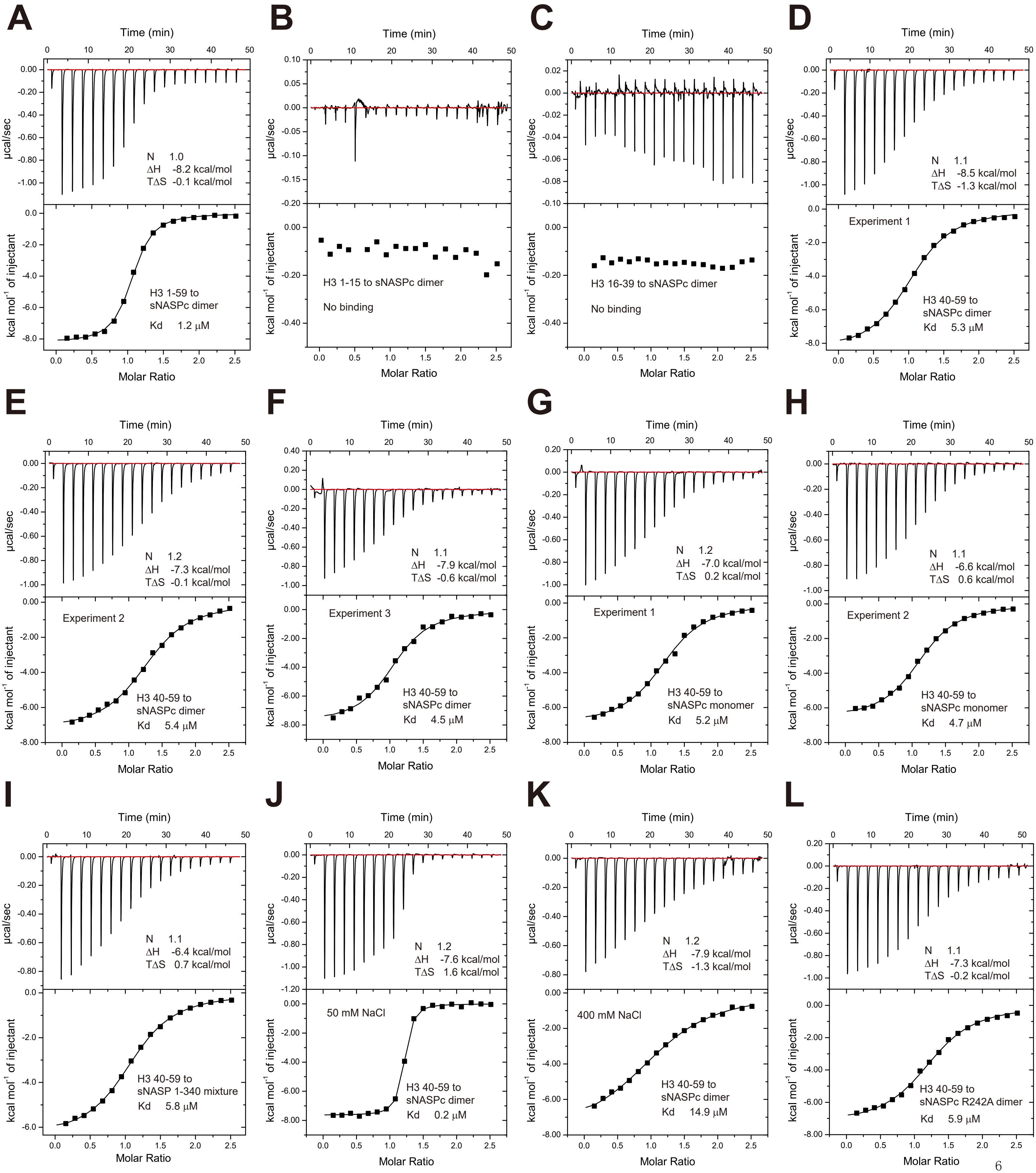
A
Time (min)
µcal/sec
N 1.0
ΔH -8.2 kcal/mol
TΔS -0.1 kcal/mol
kcal mol-1 of injectant
H3 1-59 to
sNASPc dimer
Kd 1.2 µM
Molar Ratio
B
Time (min)
µcal/sec
kcal mol-1 of injectant
H3 1-15 to sNASPc dimer
No binding
Molar Ratio
C
Time (min)
µcal/sec
kcal mol-1 of injectant
H3 16-39 to sNASPc dimer
No binding
Molar Ratio
D
Time (min)
µcal/sec
N 1.1
ΔH -8.5 kcal/mol
TΔS -1.3 kcal/mol
kcal mol-1 of injectant
Experiment 1
H3 40-59 to
sNASPc dimer
Kd 5.3 µM
Molar Ratio
E
Time (min)
µcal/sec
N 1.2
ΔH -7.3 kcal/mol
TΔS -0.1 kcal/mol
kcal mol-1 of injectant
Experiment 2
H3 40-59 to
sNASPc dimer
Kd 5.4 µM
Molar Ratio
F
Time (min)
µcal/sec
N 1.1
ΔH -7.9 kcal/mol
TΔS -0.6 kcal/mol
kcal mol-1 of injectant
Experiment 3
H3 40-59 to
sNASPc dimer
Kd 4.5 µM
Molar Ratio
G
Time (min)
µcal/sec
N 1.2
ΔH -7.0 kcal/mol
TΔS 0.2 kcal/mol
kcal mol-1 of injectant
Experiment 1
H3 40-59 to
sNASPc monomer
Kd 5.2 µM
Molar Ratio
H
Time (min)
µcal/sec
N 1.1
ΔH -6.6 kcal/mol
TΔS 0.6 kcal/mol
kcal mol-1 of injectant
Experiment 2
H3 40-59 to
sNASPc monomer
Kd 4.7 µM
Molar Ratio
I
Time (min)
µcal/sec
N 1.1
ΔH -6.4 kcal/mol
TΔS 0.7 kcal/mol
kcal mol-1 of injectant
H3 40-59 to
sNASP 1-340 mixture
Kd 5.8 µM
Molar Ratio
J
Time (min)
µcal/sec
N 1.2
ΔH -7.6 kcal/mol
TΔS 1.6 kcal/mol
kcal mol-1 of injectant
50 mM NaCl
H3 40-59 to
sNASPc dimer
Kd 0.2 µM
Molar Ratio
K
Time (min)
µcal/sec
N 1.2
ΔH -7.9 kcal/mol
TΔS -1.3 kcal/mol
kcal mol-1 of injectant
400 mM NaCl
H3 40-59 to
sNASPc dimer
Kd 14.9 µM
Molar Ratio
L
Time (min)
µcal/sec
N 1.1
ΔH -7.3 kcal/mol
TΔS -0.2 kcal/mol
kcal mol-1 of injectant
H3 40-59 to
sNASPc R242A dimer
Kd 5.9 µM
Molar Ratio

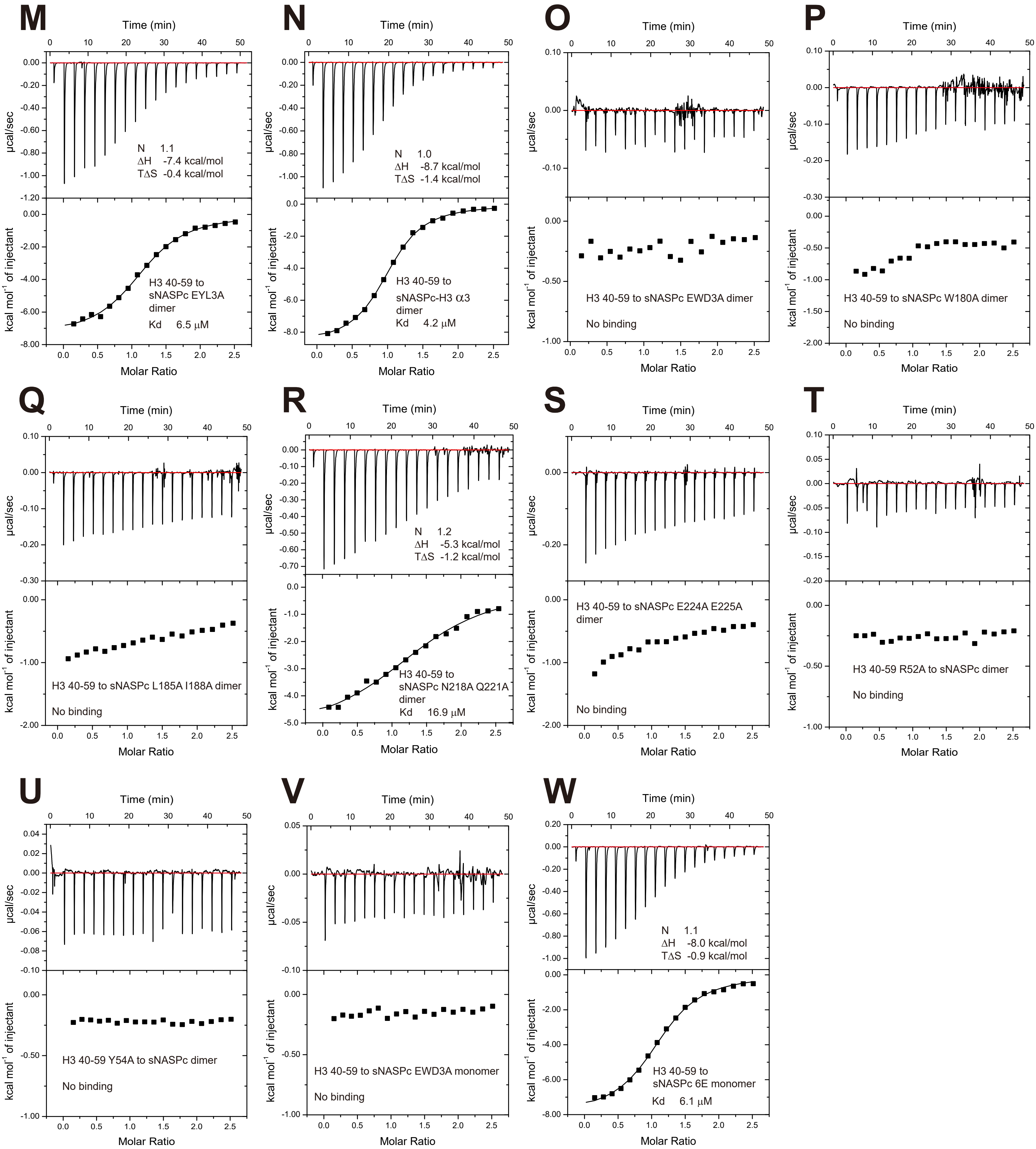

M
Time (min)
μcal/sec
kcal mol-1 of injectant
Molar Ratio
N 1.1
ΔH -7.4 kcal/mol
TΔS -0.4 kcal/mol
H3 40-59 to sNASPc EYL3A dimer
Kd 6.5 μM
N
N 1.0
ΔH -8.7 kcal/mol
TΔS -1.4 kcal/mol
H3 40-59 to sNASPc-H3 α3 dimer
Kd 4.2 μM
O
H3 40-59 to sNASPc EWD3A dimer
No binding
P
H3 40-59 to sNASPc W180A dimer
No binding
Q
H3 40-59 to sNASPc L185A I188A dimer
No binding
R
N 1.2
ΔH -5.3 kcal/mol
TΔS -1.2 kcal/mol
H3 40-59 to sNASPc N218A Q221A dimer
Kd 16.9 μM
S
H3 40-59 to sNASPc E224A E225A dimer
No binding
T
H3 40-59 R52A to sNASPc dimer
No binding
U
H3 40-59 Y54A to sNASPc dimer
No binding
V
H3 40-59 to sNASPc EWD3A monomer
No binding
W
N 1.1
ΔH -8.0 kcal/mol
TΔS -0.9 kcal/mol
H3 40-59 to sNASPc 6E monomer
Kd 6.1 μM

Supplementary Figure S3. ITC raw data. (**A-W**) The raw data of ITC assays of different sNASP constructs titrated with the N-ter fragments of H3.

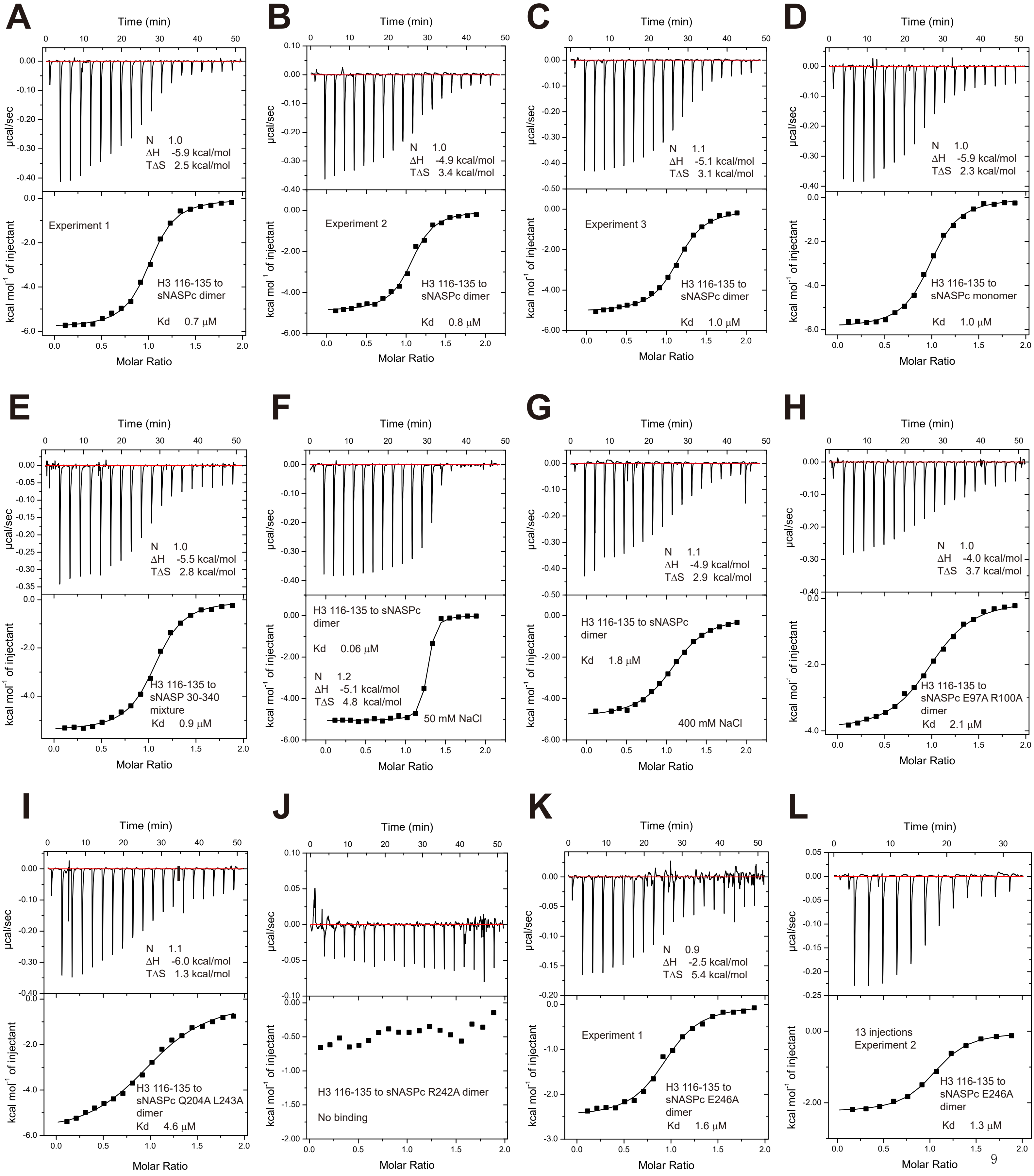
A
Time (min)
µcal/sec
N 1.0
ΔH -5.9 kcal/mol
TΔS 2.5 kcal/mol
Experiment 1
H3 116-135 to sNASPc dimer
Kd 0.7 µM
kcal mol-1 of injectant
Molar Ratio
B
N 1.0
ΔH -4.9 kcal/mol
TΔS 3.4 kcal/mol
Experiment 2
H3 116-135 to sNASPc dimer
Kd 0.8 µM
C
N 1.1
ΔH -5.1 kcal/mol
TΔS 3.1 kcal/mol
Experiment 3
H3 116-135 to sNASPc dimer
Kd 1.0 µM
D
N 1.0
ΔH -5.9 kcal/mol
TΔS 2.3 kcal/mol
H3 116-135 to sNASPc monomer
Kd 1.0 µM
E
N 1.0
ΔH -5.5 kcal/mol
TΔS 2.8 kcal/mol
H3 116-135 to sNASP 30-340 mixture
Kd 0.9 µM
F
H3 116-135 to sNASPc dimer
Kd 0.06 µM
N 1.2
ΔH -5.1 kcal/mol
TΔS 4.8 kcal/mol
50 mM NaCl
G
N 1.1
ΔH -4.9 kcal/mol
TΔS 2.9 kcal/mol
H3 116-135 to sNASPc dimer
Kd 1.8 µM
400 mM NaCl
H
N 1.0
ΔH -4.0 kcal/mol
TΔS 3.7 kcal/mol
H3 116-135 to sNASPc E97A R100A dimer
Kd 2.1 µM
I
N 1.1
ΔH -6.0 kcal/mol
TΔS 1.3 kcal/mol
H3 116-135 to sNASPc Q204A L243A dimer
Kd 4.6 µM
J
H3 116-135 to sNASPc R242A dimer
No binding
K
N 0.9
ΔH -2.5 kcal/mol
TΔS 5.4 kcal/mol
Experiment 1
H3 116-135 to sNASPc E246A dimer
Kd 1.6 µM
L
13 injections
Experiment 2
H3 116-135 to sNASPc E246A dimer
Kd 1.3 µM

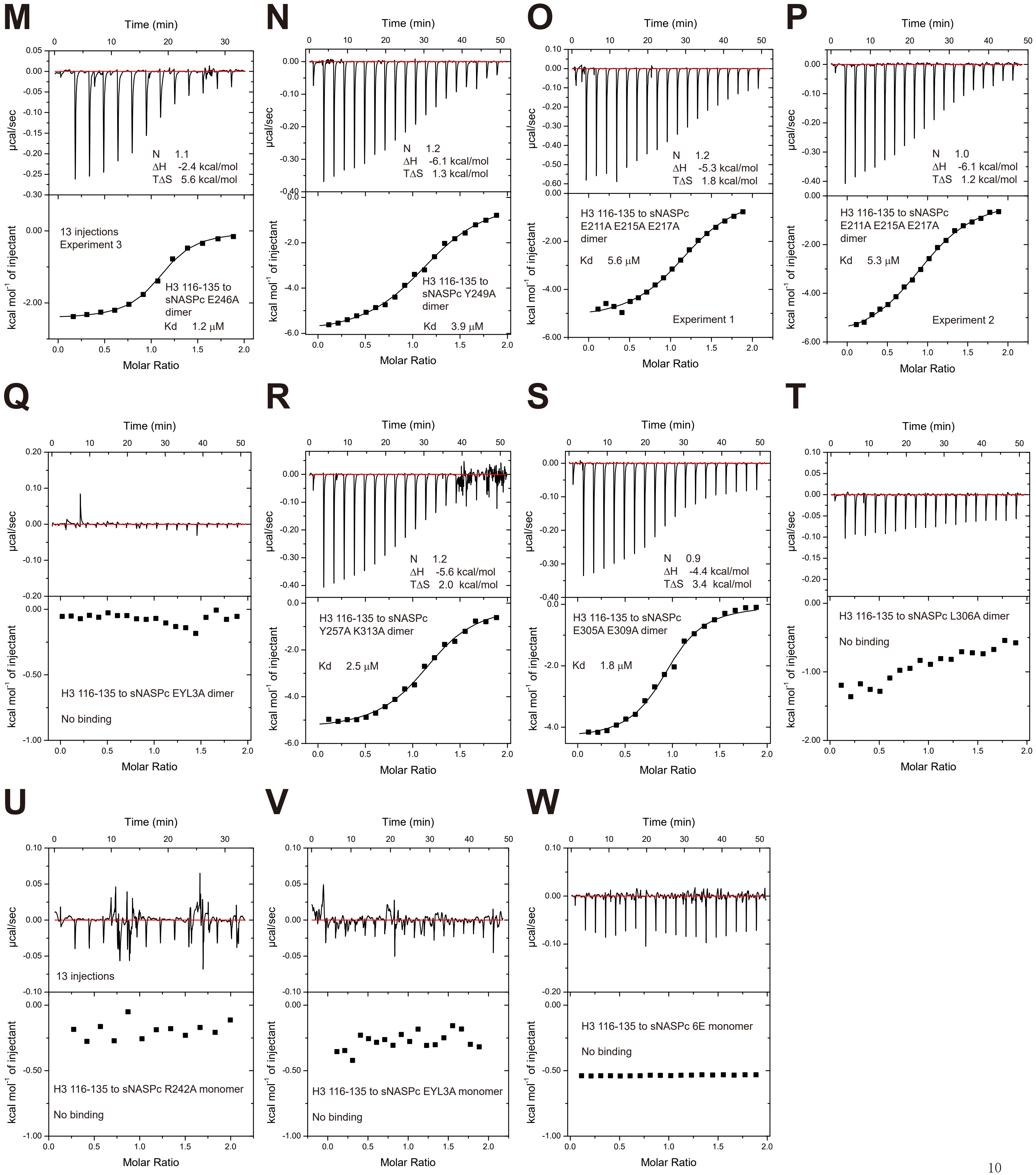
M
N 1.1
ΔH -2.4 kcal/mol
TΔS 5.6 kcal/mol
13 injections
Experiment 3
H3 116-135 to sNASPc E246A dimer
Kd 1.2 μM
N
N 1.2
ΔH -6.1 kcal/mol
TΔS 1.3 kcal/mol
H3 116-135 to sNASPc Y249A dimer
Kd 3.9 μM
O
N 1.2
ΔH -5.3 kcal/mol
TΔS 1.8 kcal/mol
H3 116-135 to sNASPc E211A E215A E217A dimer
Kd 5.6 μM
Experiment 1
P
N 1.0
ΔH -6.1 kcal/mol
TΔS 1.2 kcal/mol
H3 116-135 to sNASPc E211A E215A E217A dimer
Kd 5.3 μM
Experiment 2
Q
H3 116-135 to sNASPc EYL3A dimer
No binding
R
N 1.2
ΔH -5.6 kcal/mol
TΔS 2.0 kcal/mol
H3 116-135 to sNASPc Y257A K313A dimer
Kd 2.5 μM
S
N 0.9
ΔH -4.4 kcal/mol
TΔS 3.4 kcal/mol
H3 116-135 to sNASPc E305A E309A dimer
Kd 1.8 μM
T
H3 116-135 to sNASPc L306A dimer
No binding
U
13 injections
H3 116-135 to sNASPc R242A monomer
No binding
V
H3 116-135 to sNASPc EYL3A monomer
No binding
W
H3 116-135 to sNASPc 6E monomer
No binding
Time (min)
μcal/sec
kcal mol-1 of injectant
Molar Ratio

Supplementary Figure S4. ITC raw data. (**A-W**) The raw data of ITC assays of different sNASP constructs titrated with the C-ter fragments of H3.

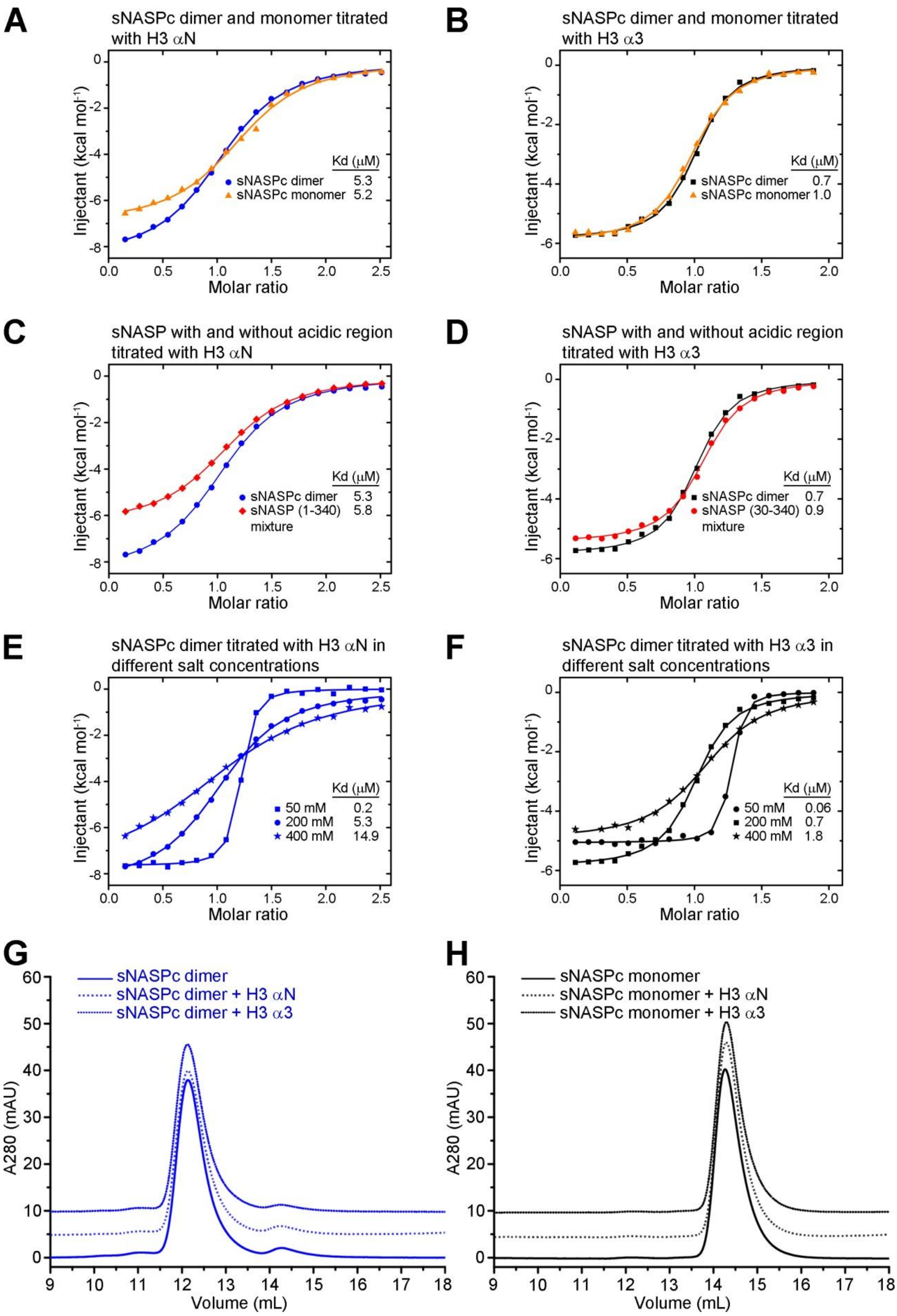

Supplementary Figure S5. ITC assays and gel-filtration analysis of the sNASPc dimer and monomer. (**A**) ITC analysis of the sNASPc dimer and

monomer titrated with the H3 αN peptide. The buffer for ITC is 50 mM Tris pH 7.5, 200 mM NaCl. The thermodynamic parameters of the ITC assays are listed in the Supplementary Table S3. All raw data from the ITC assays are shown in the Supplementary Figure S3. (**B**) ITC analysis of the sNASPc dimer and monomer titrated with the H3 α3 peptide. The buffer for ITC is 50 mM Tris pH 7.5, 200 mM NaCl. The thermodynamic parameters of the ITC assays are listed in the Supplementary Table S4. All raw data from the ITC assays are shown in the Supplementary Figure S4. (**C**) ITC analysis of sNASP with and without the acidic region titrated with the H3 αN peptide. The protein sNASP (1-340) used for ITC was a mixture of dimer and monomer. The buffer for ITC is 50 mM Tris pH 7.5, 200 mM NaCl. The thermodynamic parameters of the ITC assays are listed in the Supplementary Table S2. All raw data from the ITC assays are shown in the Supplementary Figure S3. (**D**) ITC analysis of sNASP with and without the acidic region titrated with the H3 α3 peptide. The protein sNASP (30-340) used for ITC was a mixture of dimer and monomer. The buffer for ITC is 50 mM Tris pH 7.5, 200 mM NaCl. The thermodynamic parameters of the ITC assays are listed in the Supplementary Table S3. All raw data from the ITC assays are shown in the Supplementary Figure S4. (**E**) ITC analysis of the sNASPc dimer titrated with the H3 αN peptide in different salt concentrations, including 50, 200 and 400 mM NaCl, respectively. The thermodynamic parameters of the ITC assays are listed in the Supplementary Table S2. All raw data from the ITC assays are shown in the Supplementary Figure S3. (**F**) ITC analysis of the sNASPc dimer titrated with the H3 α3 peptide in different salt concentrations, including 50, 200 and 400 mM NaCl, respectively. The thermodynamic parameters of the ITC assays are listed in the Supplementary Table S3. All raw data from the ITC assays are shown in the Supplementary Figure S4. (**G**) Gel-filtration assays of the sNASPc dimer in the presence of excess H3 αN and α3 peptides. The assays were run with the buffer of 50 mM Tris pH 7.5, 0.2 M NaCl. For clarification, the curves 'sNASPc dimer + H3 αN' and 'sNASPc dimer + H3 α3' had a vertical shift of 5 and 10

y-values, respectively. (**H**) Gel-filtration assays of the sNASPc monomer in the presence of excess H3 αN and α3 peptides. The assays were run with the buffer of 50 mM Tris pH 7.5, 0.2 M NaCl. For clarification, the curves 'sNASPc monomer + H3 αN' and 'sNASPc monomer + H3 α3' had a vertical shift of 5 and 10 y-values, respectively.

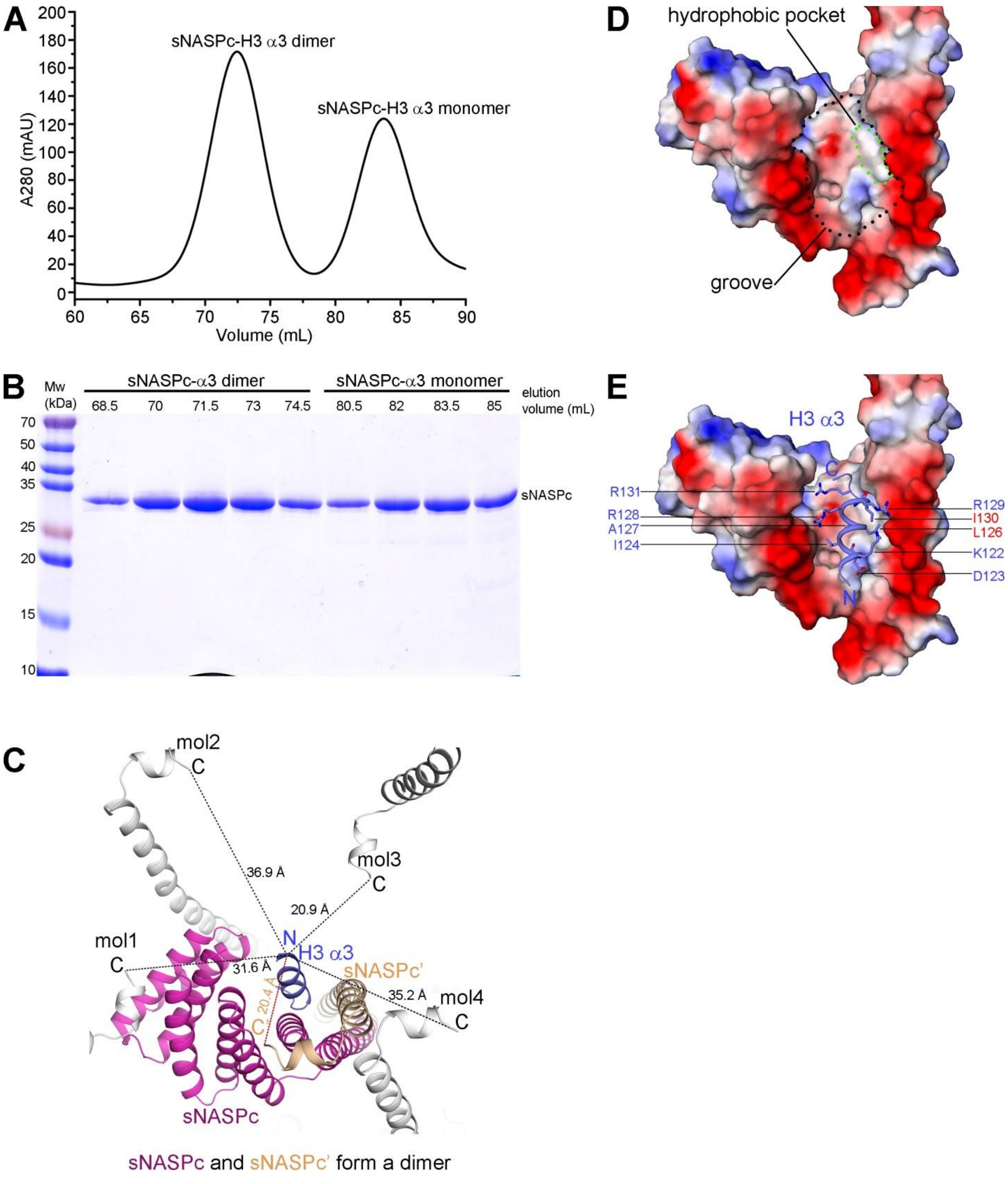

Supplementary Figure S6. Characteristics of the sNASPc-H3 α3 dimer (**A**) The sNASPc-H3 α3 cassette, made by fusion of sNASPc and the H3.3 α3 (a.a. 116–135) epitope without extra linker residues, was expressed and purified from E. coli. The resulting sNASPc-H3 α3 covalent complex showed two peaks corresponding to the dimer and monomer conformations (hereafter

referred to as sNASPc-H3 α3 dimer and monomer, respectively) on the chromatogram during gel-filtration purification. The purification used a HiLoad 16/600 Superdex 200 column pre-equilibrated with a buffer of 20 mM Tris pH7.5, 0.5 M NaCl. (**B**) The peak fractions from panel A, corresponding to the sNASPc-H3 α3 dimer and monomer, were analyzed with SDS-PAGE. The fractions were collected 1.5 mL per tube, while the numbers on top of the SDS-PAGE gel indicated the elution volume in which the fractions started. The pure fractions of dimer and monomer were pooled and concentrated for crystallization, respectively. (**C**) Analysis of the crystal lattice of the sNASPC-H3 α3 dimer structure. The sNASPc (a.a. 30–340, with a deletion Δ101–159) and H3.3 α3 (a.a. 116–135) fragment were fused together to form the sNASPc-H3 α3 fusion protein. After solving the structure of the sNASPC-H3 α3 dimer, the residues 333–340 of sNASPc and 116–119 of H3 α3 (thus totally 12 residues within the linker), are disordered and cannot be resolved in the density map. The 12-residue linker can cover a distance about 43 Å. The protomers sNASPc (magenta color) and sNASPc' (wheat color) form a dimer. The H3 α3 molecule bound by the protomer sNASPc seems from the H3 α3 fragment fused with the other protomer sNASPc', as the distance between the sNASPc' C'-terminus and H3 α3 N-terminus is 20.4 Å. We cannot exclude the possibility that the H3 α3 molecule is from the other nearby sNASPc molecules in the crystal lattice, such as the C-termini of the molecules 1, 2, 3 or 4 have distances to the H3 α3 N-terminus within 43 Å. (**D-E**) Surface view of sNASPc color-coded with the electrostatic potential (red, negatively charged; blue, positively charged). The H3 α3-binding groove and the hydrophobic pocket within the groove are highlighted with black and green dashed circles, respectively (**D**). The H3 α3 peptide bound in the groove is shown as ribbon representation (**E**); and the interacting residues of H3 α3 are shown in sticks representation (**E**). The side chains of H3 Leu126 and Ile130 (labeled in red) are bound into the hydrophobic pocket.

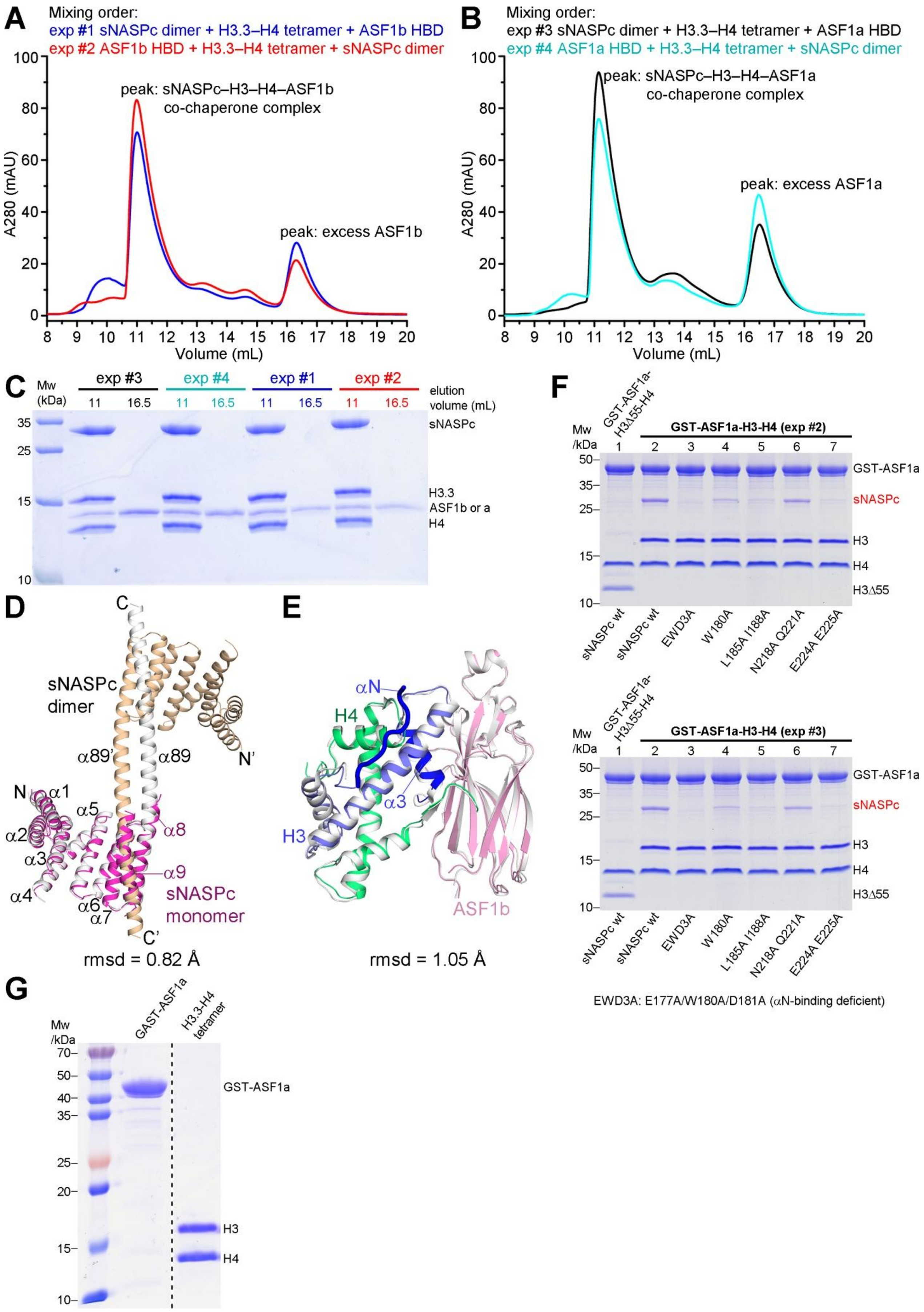
A
Mixing order:
exp #1 sNASPc dimer + H3.3–H4 tetramer + ASF1b HBD
exp #2 ASF1b HBD + H3.3–H4 tetramer + sNASPc dimer
peak: sNASPc–H3–H4–ASF1b co-chaperone complex
peak: excess ASF1b
A280 (mAU)
Volume (mL)
B
Mixing order:
exp #3 sNASPc dimer + H3.3–H4 tetramer + ASF1a HBD
exp #4 ASF1a HBD + H3.3–H4 tetramer + sNASPc dimer
peak: sNASPc–H3–H4–ASF1a co-chaperone complex
peak: excess ASF1a
A280 (mAU)
Volume (mL)
C
Mw (kDa)
exp #3
exp #4
exp #1
exp #2
elution volume (mL)
sNASPc
H3.3
ASF1b or a
H4
D
sNASPc dimer
sNASPc monomer
rmsd = 0.82 Å
E
H4
H3
ASF1b
rmsd = 1.05 Å
F
Mw /kDa
GST-ASF1a-H3Δ55-H4
GST-ASF1a-H3-H4 (exp #2)
GST-ASF1a
sNASPc
H3
H4
H3Δ55
sNASPc wt
sNASPc wt
EWD3A
W180A
L185A I188A
N218A Q221A
E224A E225A
GST-ASF1a-H3-H4 (exp #3)
EWD3A: E177A/W180A/D181A (αN-binding deficient)
G
Mw /kDa
GAST-ASF1a
H3.3-H4 tetramer
GST-ASF1a
H3
H4

Supplementary Figure S7. Characteristics of the sNASPc-8G-ASF1b–H3–H4 heterotetramer. (**A**) Reconstitution of the sNASPc–H3–H4–ASF1b co-chaperone complex. For exp #1, the purified sNASPc dimer was mixed with the H3–H4 tetramer first, and then with ASF1b (a.a. 1–158) at a molar ratio of 1:1:2.4. For exp #2, ASF1b (a.a. 1–158) was mixed with the H3–H4 tetramer first, and then with the sNASPc dimer at a molar ratio of 2.4:1:1. The mixed samples were respectively applied to a Superdex 200 Increase 10/300 GL column (GE Healthcare) pre-equilibrated with a buffer of 20 mM Tris pH 7.5, 0.5 M NaCl. (**B**) Reconstitution of the sNASPc–H3–H4–ASF1a co-chaperone complex. The same experimental settings as in panel A were repeated for exp #3 and #4, with proteins sNASPc dimer, H3–H4 tetramer and ASF1a (a.a. 1-155). From panels A and B, we noted that the reconstitutions were not dependent on the mixing order of the sub-components. The collected peak fractions from panels A and B were analyzed with SDS-PAGE in panel C. To mesure the molar masses of the reconstituted sNASPc–H3–H4–ASF1b and sNASPc–H3–H4–ASF1a co-chaperone complexes, the corresponding peak fractions were collected and concentrated for the SEC-MALS experiments, of which the results were shown in Figure 3A. (**C**) The peaks fractions coresponding to the sNASPc–H3–H4–ASF1b co-chaperone complex and excess ASF1b (exp #1 and #2), and to the sNASPc–H3–H4–ASF1a co-chaperone complex and excess ASF1a (exp #3 and #4) were analyzed by SDS-PAGE. (**D**) Superimposition of the structure of the sNASPc monomer (colored in magenta) from the sNASPc-8G-ASF1b–H3–H4 heterotetramer onto the structure of the sNASPc dimer (two protomers in white and wheat, respectively). The rmsd of the two superimposed structures is 0.82 Å. (**E**) Superimposition of the structure of the H3–H4–ASF1b part (H3, H4 and ASF1b, are colored with blue, green and pink, respectively) derived from the sNASPc-8G-ASF1b–H3–H4 heterotetramer onto the known structure of the ASF1a–H3–H4 trimer (colored in white; PDB 2IO5). The H3 αN and α3 regions of the H3–H4–ASF1b part are highlighted in dark blue. The rmsd of the

two superimposed structures is 1.05 Å. (**F**) The biological replicates (exp #2 and #3) for Figure 3F. Pulldowns of the sNASPc dimer and its mutants (dimers) in the αN-binding site using the GST-ASF1a–H3–H4 or GST-ASF1a–H3Δ55–H4 complexes. H3Δ55 indicates H3 with a deletion of the first 55 residues, including the H3 αN region. The results in Figure 3F (exp #1) and panel F (exp #2 and #3) are highly consistent. (**G**) The purities of the GST-ASF1a (full-length) and H3.3–H4 tetramer used to reconstitute the GST-ASF1a–H3–H4 complex in panel F and the Figure 3F and 3G were checked by SDS-PAGE.

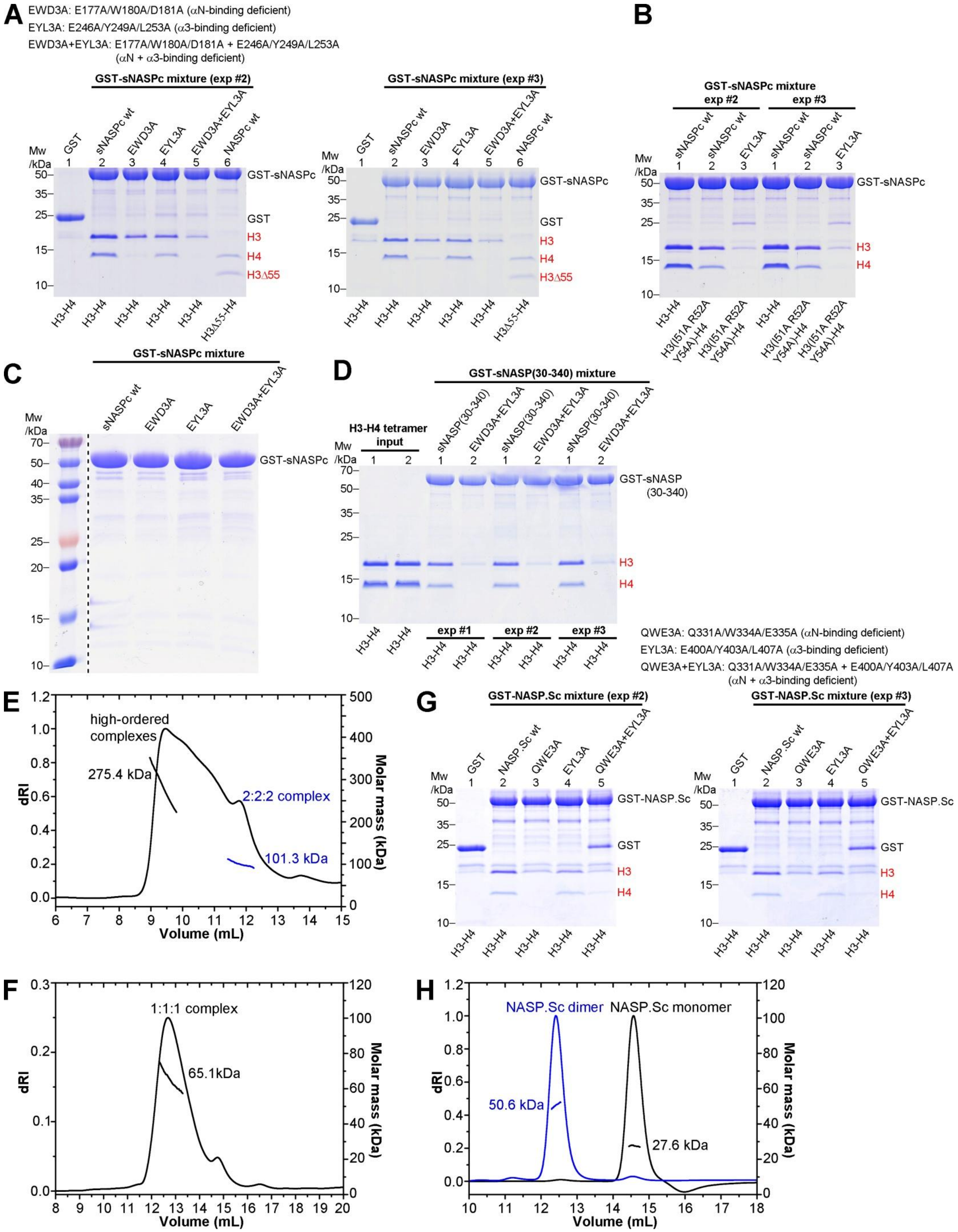
A
EWD3A: E177A/W180A/D181A (αN-binding deficient)
EYL3A: E246A/Y249A/L253A (α3-binding deficient)
EWD3A+EYL3A: E177A/W180A/D181A + E246A/Y249A/L253A
(αN + α3-binding deficient)
GST-sNASPc mixture (exp #2)
GST-sNASPc mixture (exp #3)
Mw /kDa
GST
sNASPc wt
EWD3A
EYL3A
EWD3A+EYL3A
NASPc wt
GST-sNASPc
GST
H3
H4
H3Δ55
H3-H4
H3Δ55-H4
B
GST-sNASPc mixture
exp #2
exp #3
H3(I51A R52A Y54A)-H4
C
GST-sNASPc mixture
D
GST-sNASP(30-340) mixture
H3-H4 tetramer input
sNASP(30-340)
GST-sNASP (30-340)
exp #1
exp #2
exp #3
QWE3A: Q331A/W334A/E335A (αN-binding deficient)
EYL3A: E400A/Y403A/L407A (α3-binding deficient)
QWE3A+EYL3A: Q331A/W334A/E335A + E400A/Y403A/L407A
(αN + α3-binding deficient)
E
high-ordered complexes
275.4 kDa
2:2:2 complex
101.3 kDa
dRI
Molar mass (kDa)
Volume (mL)
F
1:1:1 complex
65.1kDa
G
GST-NASP.Sc mixture (exp #2)
GST-NASP.Sc mixture (exp #3)
NASP.Sc wt
QWE3A
GST-NASP.Sc
H
NASP.Sc dimer
NASP.Sc monomer
50.6 kDa
27.6 kDa

Supplementary Figure S8. Interactions between sNASPc and H3–H4 dimers. (**A**) The biological replicates (exp #2 and #3) for Figure 4A. Pulldowns of GST-tagged sNASPc and its mutants with the H3–H4 tetramer or the H3Δ55–H4 tetramer. H3Δ55 indicates H3 with a deletion of the first 55 residues, lacking the H3 αN region. As mentioned in methods the GST-tagged sNASPc and its mutants could not be separated into dimer and monomer peaks during the purification steps, the conformations of these proteins used for GST pulldowns were mixtures of dimer and monomer (referred to as GST-sNASPc mixture). The pulldowns were done by mixing the GST-sNASPc mixture and its mutants with excess H3.3–H4 tetramer or H3.3Δ55–H4 tetramer in the incubation buffer of 20 mM Tris pH 7.5, 0.3 M NaCl, and by washing with the washing buffer of 20 mM Tris pH 7.5, 0.75 M NaCl, 0.5% v/v Triton X-100. Then all the samples were analyzed with SDS-PAGE. For convenience, the mutants are listed in the figure. The results in Figure 4A (exp #1) and panel A (exp #2 and #3) are highly consistent. (**B**) The biological replicates (exp #2 and #3) for Figure 4B. Pulldowns of GST-sNASPc mixture and its mutants with the H3–H4 tetramer or the H3 (I51A R52A Y54A)–H4 tetramer. H3 (I51A R52A Y54A) is a mutant with triple mutations on the H3 αN region. The pulldowns were done in the same way as panel A. The results in Figure 4B (exp #1) and panel B (exp #2 and #3) are highly consistent. (**C**) The purities of the GST-tagged sNASPc and its mutants used for pulldowns in panels A and B, and the Figure 4A and 4B were checked by SDS-PAGE. (**D**) Pulldowns of GST-sNASP$^{(30\text{-}340)}$ mixture and its mutant EWD3A+EYL3A with the H3–H4 tetramer. The pulldowns were done in the same way as panel A and with 3 biological replicates (exp #1, #2 and #3). (**E**) SEC-MALS analysis of the sNASPc dimer–H3–H4 complex. The purified sNASPc dimer was mixed with the H3–H4 tetramer at a molar ratio of 1:1, which was then applied to the SEC-MALS assay with a running buffer of 20 mM Tris pH 7.5, 0.5 M NaCl. The measured mass and expected mass are compared, as shown in Supplementary Table S1. (**F**) SEC-MALS analysis of the sNASPc

monomer–H3–H4 complex. The purified sNASPc monomer was mixed with the H3–H4 tetramer at a molar ratio of 1:0.5, which was then applied to the SEC-MALS assay with a running buffer of 20 mM Tris pH 7.5, 0.5 M NaCl. The measured mass and expected mass are compared, as shown in Supplementary Table S1. (**G**) The biological replicates (exp #2 and #3) for Figure 4D. Pulldowns of GST-NASP.Sc mixture and its mutants with the H3–H4 tetramer. NASP.Sc is 'NASP.S core', containing residues 23–495 with the acidic region (a.a. 97–314) deleted. For convenience, the mutants are listed in the figure. The results in Figure 4D (exp #1) and panel F (exp #2 and #3) are highly consistent. (**H**) SEC-MALS analysis of the NASP.Sc dimer and monomer. The measured mass and expected mass are compared, as shown in Supplementary Table S1.

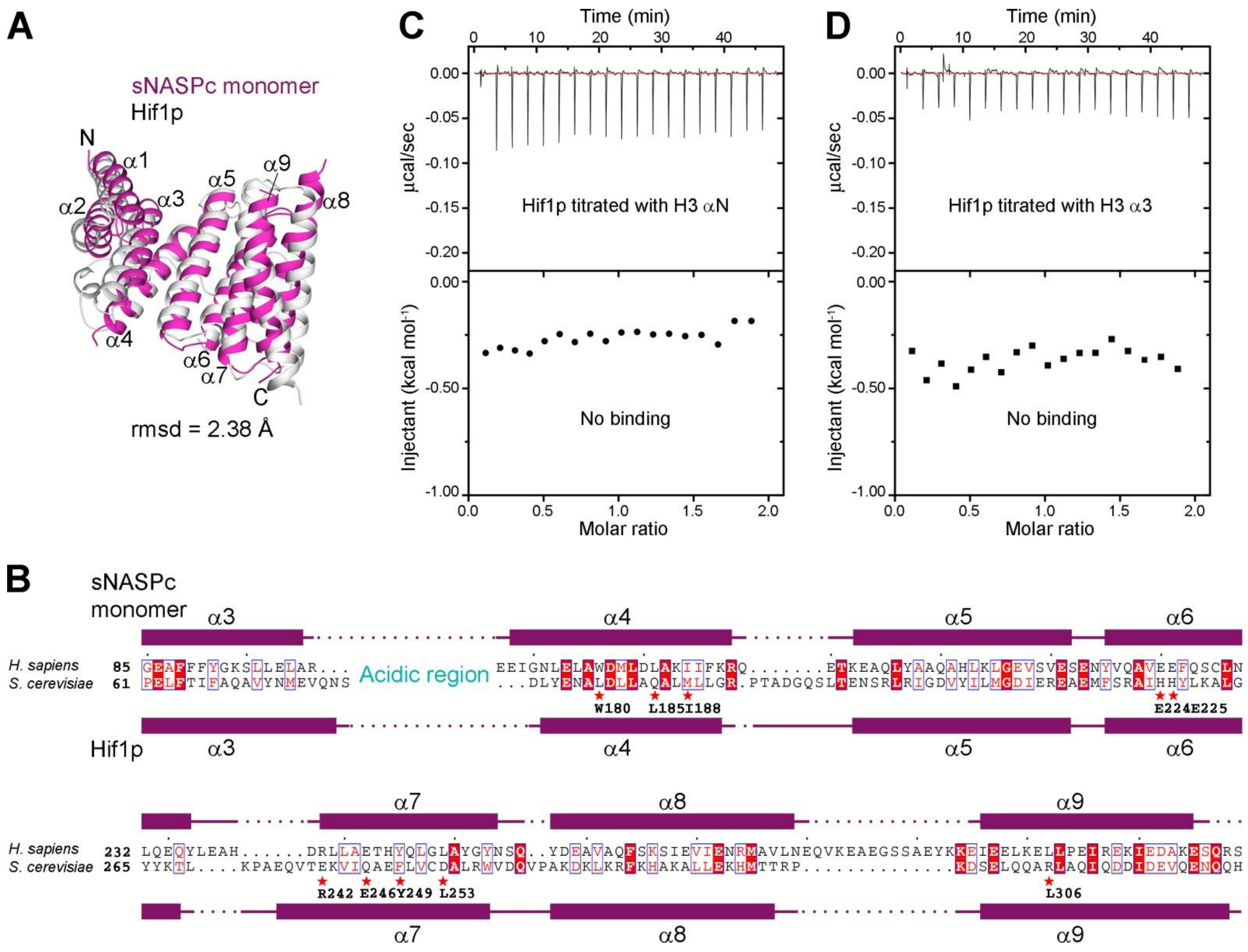

Supplementary Figure S9. Comparisons of sNASP and budding yeast Hif1p. (**A**) Superimposition of the structure of the sNASPc monomer (colored in magenta) derived from the sNASPc-8G-ASF1b–H3–H4 heterotetramer onto the structure of budding yeast Hif1p (colored in white; PDB 4NQ0). The superimposition covered the TPR2-4 motifs and the capping helices of sNASPc and Hif1p, whilst the TPR1 motifs did not fit well in the two structures. The rmsd of the two superimposed structures is 2.38 Å. (**B**) Sequence alignment of *H. sapiens* sNASP (NP_689511) and *S. cerevisae* Hif1p (NP_013078). The alignment was focus on the TPR2-4 motifs and the capping helices, whilst the TPR1 motifs and the acidic regions were not conserved and omitted for alignment. The alignment was manually adjusted based on

structural superimposition in panel A. The conserved and identical residues are boxed and highlighted in red. Secondary structure elements derived from the structures of the sNASPc monomer and budding yeast Hif1p (PDB 4NQ0) are shown on top and bottom of the alignment, respectively. Under the alignments, '★' highlights the key residues of sNASP in the H3 αN-binding site (W180, L185, I188, E224 and E225) and H3 α3-binding site (R242, E246, Y249, L253 and L306), illustrating that some of these key residues are not conserved in Hif1p. (**C**) ITC analysis of Hif1p titrated with the H3 αN peptide, which did not show any obvious interaction. (**D**) ITC analysis of Hif1p titrated with the H3 α3 peptide, which did not show any obvious interaction. This confirmed the result from Bowman et al (1).

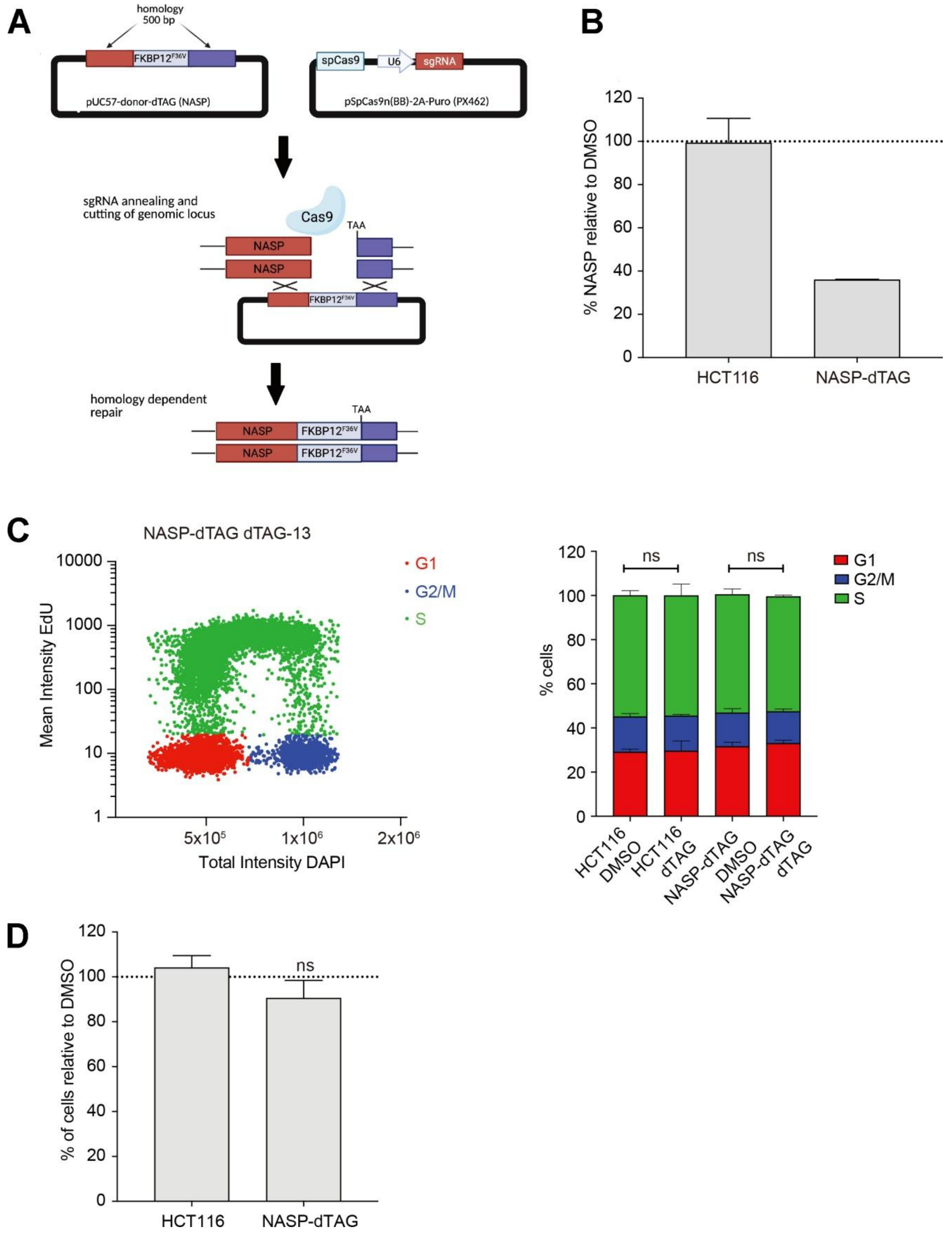

Supplementary Figure S10. Characterization of NASP-dTAG cell line. (**A**) Schematic depiction of the $FKBP12^{F36V}$ knock-in strategy. (**B**) High-content microscopy of HCT116 wt and NASP-dTAG cells treated with DMSO or dTAG-13 for 48 hours and pulsed with EdU before fixation. The NASP signal

was measured in the nucleus and shown relative to the DMSO control. Bars represent the mean with s.d. (n=3). (**C**) Cell cycle distribution measured by high-content microscopy in cells treated as in panel b. (left panel) Representative diagram illustrating gating strategy for quantification of G1, S and G2/M populations. (right panel) Bar-diagram showing cell cycle distribution across HCT116 wt and NASP-dTAG cells treated as indicated. Mean is shown with ± s.d (n=3). ns, non-significant indicates P>0.05 in multiple unpaired t test (from left, G1 P= 0.873142; 0.349609; S phase P=0.919112; 0.550069; G2 P=0.908600; 0.366155). (**D**) Cell viability measured by cell titer blue in HCT116 wt and NASP-dTAG cells treated with DMSO or dTAG-13 for 48 hours. Viability is shown relative to the DMSO control with bars indicating the mean with s.d. (n=3). ns, non-significant indicates P>0.05 in multiple unpaired t test (from left to right, P= 0.837880; 0. 0.311381)

Supplementary Table S1. Results of SEC-MALS assays.

Complex	Expected stoichiometry	Expected mass (kDa)	Measured mass* (kDa)	Measured mass/Expected mass
sNASPc dimer	2	56.4	59.1	1.05
sNASPc monomer	1	28.2	25.7	0.92
sNASPc 6E mutant	1	28.3	30.2	1.07
sNASPc–H3–H4–ASF1a(1-155)	1:1:1:1	72.1	76.8	1.07
sNASPc–H3–H4–ASF1b(1-158)	1:1:1:1	72.5	74.6	1.03
sNASPc-8G-ASF1b(1-158)–H3–H4	1:1:1	71.1	70.6	0.99
sNASPc dimer–H3–H4 complex	2:2:2 complex	109.2	101.3	0.93
	2×(2:2:2) or 3×(2:2:2) (higher-order complexes)	218.4 or 327.6	275.4	1.26 or 0.84
sNASPc monomer–H3–H4 complex	1:1:1 complex	54.6	65.1	1.19
frog NASP.Sc dimer	2	57.2	50.6	0.88
frog NASP.Sc monomer	1	28.6	27.6	0.97

* Buffer conditions: 20 mM Tris pH 7.5, 0.5 M NaCl.

Supplementary Table S2. Protein stability parameters of different sNASPc proteins. The stabilities of differnerent sNASPc consutructs and mutants were evaluated through the inflection temperatures (Ti), which were determined by using Tycho NT.6 (NanoTemper Technologies). The proteins were diluted into 0.1 mg/mL with 1 X PBS buffer (10 mM Na_2HPO_4, 1.75 mM KH_2PO_4, 137 mM NaCl, 2.65 mM KCl, pH 7.4). n=3 independent experiments, error bars represent mean ± SD.

Protein	Inflection temperature (T_i; °C)	Initial ratio	Δ ratio	Sample brightness
sNASPc dimer	**58.9 ± 0.1**	1.093 ± 0.002	-0.367 ± 0.005	32.4 ± 0.4
sNASPc monomer	**58.8 ± 0.3**	1.112 ± 0.012	-0.393 ± 0.008	32.8 ± 1.6
sNASPc 6E mutant[a]	**38.9 ± 0.1**	0.943 ± 0.003	-0.210 ± 0.004	30.6 ± 0.5
sNASPc R242A dimer	**55.4 ± 0.2**	1.098 ± 0.005	-0.367 ± 0.015	19.5 ± 0.1
sNASPc EYL3A dimer[b]	**48.8 ± 0.1**	1.064 ± 0.001	-0.273 ± 0.002	38.7 ± 0.8
sNASPc L306A dimer	**54.7 ± 0.1**	1.099 ± 0.002	-0.387 ± 0.002	20.2 ± 0.2
sNASPc EWD3A dimer[c]	**61.6 ± 0.3**	0.315 ± 0.027	0.075 ± 0.017	8.0 ± 0.6
sNASPc L185A I188A dimer	**60.4 ± 0.1**	1.037 ± 0.009	-0.310 ± 0.015	17.2 ± 0.7
sNASPc E224A E225A dimer	**54.1 ± 0.1**	1.036 ± 0.003	-0.318 ± 0.001	34.1 ± 0.5

[a] 6E is for V265E I272E L282E V286E I300E L307E;
[b] EYL3A is for E246A Y249A L253A;
[c] EWD3A is for E177A W180A D181A.
Note: The mutants R242A, EYL3A and L306A are H3 α3-binding deficient, whereas the mutants EWD3A, L185A I188A, and E224A E225A are H3 αN-binding deficient.

Supplementary Table S3. Thermodynamic parameters of different sNASP constructs titrated with the N-ter fragments of H3. Most of the ITC experiments were carried out at 25 ℃ with a buffer of 50 mM Tris pH 7.5, 200 mM NaCl, unless otherwise specified. The H3 N-ter peptide in syringe is 0.8 mM, and the sNASP protein in cell is 0.06 mM (counted as sNASP monomer). The data were processed with Microcal Origin software and the curves were fit to the 'one set of sites' model.

Protein	Peptide	ΔH kcal/mol	TΔS kcal/mol	*K*d μM	N
sNASPc dimer	H3 (1–59)	-8.2	-0.1	1.2	1.0
sNASPc dimer	H3 (1–15)			NB	
sNASPc dimer	H3 (16–39)			NB	
sNASPc dimer (exp #1)	H3 αN[a]	-8.5	-1.3	5.3	1.1
sNASPc dimer (exp #2)	H3 αN	-7.3	-0.1	5.4	1.2
sNASPc dimer (exp #3)	H3 αN	-7.9	-0.6	4.5	1.1
sNASPc monomer (exp #1)	H3 αN	-7.0	0.2	5.2	1.2
sNASPc monomer (exp #2)	H3 αN	-6.6	0.6	4.7	1.1
sNASP (1-340) mixture	H3 αN	-6.4	0.7	5.8	1.1
sNASPc dimer (50 mM NaCl buffer)	H3 αN	-7.6	1.6	0.2	1.2
sNASPc dimer (400 mM NaCl buffer)	H3 αN	-7.9	-1.3	14.9	1.2
sNASPc R242A dimer	H3 αN	-7.3	-0.2	5.9	1.1
sNASPc EYL3A dimer[b]	H3 αN	-7.4	-0.4	6.5	1.1
sNASPc-H3 α3 dimer	H3 αN	-8.7	-1.4	4.2	1.0
sNASPc EWD3A dimer[c]	H3 αN			NB	
sNASPc W180A dimer	H3 αN			NB	
sNASPc L185A I188A dimer	H3 αN			NB	
sNASPc N218A Q221A dimer	H3 αN	-5.3	1.2	16.9	1.2
sNASPc E224A E225A dimer	H3 αN			NB	
sNASPc dimer	H3 αN R52A			NB	
sNASPc dimer	H3 αN Y54A			NB	
sNASPc EWD3A monomer	H3 αN			NB	
sNASPc 6E monomer	H3 αN	-8.0	-0.9	6.1	

[a] H3 αN is for H3 (40-59);
[b] EYL3A is for E246A Y249A L253A;
[c] EWD3A is for E177A W180A D181A.
Note: the experiments for the sNASPc dimer and monomer have been repeated, with n=3 and 2 respectively.

Supplementary Table S4. Thermodynamic parameters of different sNASP constructs titrated with the C-ter fragments of H3. Most of the ITC experiments were carried out at 25 ℃ with a buffer of 50 mM Tris pH 7.5, 200 mM NaCl, unless otherwise specified. The H3 C-ter peptide in syringe is 0.4 mM, and the sNASP protein in cell is 0.04 mM (counted as sNASP monomer). The data were processed with Microcal Origin software and the curves were fit to the 'one set of sites' model.

Protein	Peptide	ΔH kcal/mol	TΔS kcal/mol	*K*d μM	N
sNASPc dimer (exp #1)	H3 α3[a]	-5.9	2.5	0.7	1.0
sNASPc dimer (exp #2)	H3 α3	-4.9	3.4	0.8	1.0
sNASPc dimer (exp #3)	H3 α3	-5.1	3.1	1.0	1.1
sNASPc monomer	H3 α3	-5.9	2.3	1.0	1.0
sNASP (30-340) mixture	H3 α3	-5.5	2.8	0.9	1.0
sNASPc dimer (50 mM NaCl buffer)	H3 α3	-5.1	4.8	0.06	1.2
sNASPc dimer (400 mM NaCl buffer)	H3 α3	-4.9	2.9	1.8	1.1
sNASPc E97A R100A dimer	H3 α3	-4.0	3.7	2.1	1.0
sNASPc Q204A L243A dimer	H3 α3	-6.0	1.3	4.6	1.1
sNASPc R242A dimer	H3 α3			NB	
sNASPc E246A dimer (exp #1)	H3 α3	-2.5	5.4	1.6	0.9
sNASPc E246A dimer (exp #2)	H3 α3	-2.3	5.8	1.3	1.0
sNASPc E246A dimer (exp #3)	H3 α3	-2.4	5.6	1.2	1.1
sNASPc Y249A dimer	H3 α3	-6.1	1.3	3.9	1.2
sNASPc EEE3A dimer (exp #1)[b]	H3 α3	-5.3	1.8	5.6	1.2
sNASPc EEE3A dimer (exp #2)	H3 α3	-6.1	1.2	5.3	1.0
sNASPc EYL3A dimer[c]	H3 α3			NB	
sNASPc Y257A K313A dimer	H3 α3	-5.6	2.0	2.5	1.2
sNASPc E305A E309A dimer	H3 α3	-4.4	3.4	1.8	0.9
sNASPc L306A dimer	H3 α3			NB	
sNASPc R242A monomer	H3 α3			NB	
sNASPc EYL3A monomer	H3 α3			NB	
sNASPc 6E monomer	H3 α3			NB	

[a] H3 α3 is for H3 (116-135);
[b] EEE3A is for E211A E215A E217A;
[c] EYL3A is for E246A Y249A L253A;
[d] EWD3A is for E177A W180A D181A.
Note: the experiments for the sNASPc dimer and the mutants E246A and EEE3A have been repeated, with n=3, 3 and 2 respectively.

Reference

1. Bowman, A., Lercher, L., Singh, H.R., Zinne, D., Timinszky, G., Carlomagno, T. and Ladurner, A.G. (2016) The histone chaperone sNASP binds a conserved peptide motif within the globular core of histone H3 through its TPR repeats. *Nucleic acids research*, **44**, 3105-3117.
